# Genome-wide association studies and prediction of 17 traits related to phenology, biomass and cell wall composition in the energy grass *Miscanthus sinensis*

**DOI:** 10.1111/nph.12621

**Published:** 2013-12-06

**Authors:** Gancho T Slavov, Rick Nipper, Paul Robson, Kerrie Farrar, Gordon G Allison, Maurice Bosch, John C Clifton-Brown, Iain S Donnison, Elaine Jensen

**Affiliations:** 1 Institute of Biological, Environmental and Rural Sciences, Aberystwyth UniversityAberystwyth, SY23 3EB, UK; 2 Floragenex Inc.Eugene, OR, 97403, USA

**Keywords:** genomic selection, genome-wide association studies (GWAS), *Miscanthus sinensis*, molecular markers, RAD-Seq, single-nucleotide variants

## Abstract

- Increasing demands for food and energy require a step change in the effectiveness, speed and flexibility of crop breeding. Therefore, the aim of this study was to assess the potential of genome-wide association studies (GWASs) and genomic selection (i.e. phenotype prediction from a genome-wide set of markers) to guide fundamental plant science and to accelerate breeding in the energy grass *Miscanthus*.
- We generated over 100 000 single-nucleotide variants (SNVs) by sequencing restriction site-associated DNA (RAD) tags in 138 *Micanthus sinensis* genotypes, and related SNVs to phenotypic data for 17 traits measured in a field trial.
- Confounding by population structure and relatedness was severe in naïve GWAS analyses, but mixed-linear models robustly controlled for these effects and allowed us to detect multiple associations that reached genome-wide significance. Genome-wide prediction accuracies tended to be moderate to high (average of 0.57), but varied dramatically across traits. As expected, predictive abilities increased linearly with the size of the mapping population, but reached a plateau when the number of markers used for prediction exceeded 10 000–20 000, and tended to decline, but remain significant, when cross-validations were performed across subpopulations.
- Our results suggest that the immediate implementation of genomic selection in *Miscanthus* breeding programs may be feasible.

## Introduction

Cost-efficient genotyping protocols based on next-generation sequencing (Davey *et al.*, 2011) have narrowed the gap between model and non-model plants, thereby creating great opportunities in crop breeding (Hamblin *et al.*, 2011; Morrell *et al.*, 2012). Two approaches that are likely to have particularly strong and widespread impacts are the dissection of complex traits through genome-wide association studies (GWASs; Cardon & Bell, 2001) and genome-wide phenotype prediction (genomic selection; Meuwissen *et al.*, 2001). By informing fundamental science and applied breeding, respectively, these two approaches have the potential to bridge the molecular and statistical void between phenotype and genotype, thereby shedding light on key biological questions.

With increasing demands for food and energy, decreasing land base and changing environmental conditions (Foley *et al.*, 2011; Valentine *et al.*, 2012), the urgency to develop accelerated crop breeding strategies cannot be overstated. The use of lignocellulosic energy crops is one of many potential mitigating factors for this problem, but the widespread adoption of these crops has been slow and hampered by a number of technological and economic challenges, with major breakthroughs required both in terms of biomass yield on low-value lands and conversion efficiency (Sims *et al.*, 2010; Feltus & Vandenbrink, 2012; Service, 2013). Genomic approaches (e.g. GWAS and genomic selection) can substantially facilitate these breakthroughs and are therefore among the focal points in this area of research (Rubin, 2008; Feltus & Vandenbrink, 2012; Slavov *et al.*, 2013a).

The tropical C_4_ grass *Miscanthus* is a promising energy crop because of its broad adaptability, potentially very high yields and low requirements for agricultural inputs (Clifton-Brown *et al.*, 2004, 2007; Hastings *et al.*, 2009). However, *Miscanthus* species are undomesticated, and several breeding programs in East Asia, Europe and North America are targeting the accelerated development of hybrids and varieties that are high yielding, can be established and maintained at low cost and have cell wall characteristics that allow efficient conversion to fuels and products. In a recent study, we used a combination of phenotypic data from a replicated field trial and 120 molecular markers to delineate an experimental population of *M. sinensis* for GWAS and genome-wide prediction (Slavov *et al.*, 2013b). This population had relatively weak substructure (*F*_ST_ < 0.06), but captured high levels of genetic variation across a range of phenotypic traits related to phenology, biomass productivity and cell wall composition.

Building on this study, we: (1) sequenced restriction site-associated DNA (RAD) tags in 142 *M. sinensis* genotypes and generated over 100 000 ‘RAD-Seq' single-nucleotide variants (SNVs); (2) confirmed patterns of putatively neutral population structure detected in our previous study, but also used the power of the RAD-Seq markers to add substantial new details, including the hypothetical geographical origins for a large number of accessions with unknown sampling locations; and (3) assessed the potential of GWASs and genome-wide prediction to guide biological discovery and accelerated breeding in *Miscanthus*.

## Materials and Methods

### Plant materials, phenotyping and DNA extraction

**Fig 1:**
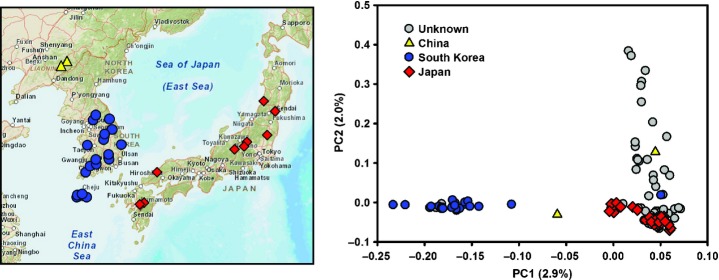
Geographical distribution and principal component (PC) analysis of population genetic structure for 138 *Miscanthus sinensis* genotypes using 14 073 single-nucleotide variant loci. The percentages of the total variation explained by each PC are shown in parentheses.

**Fig 2:**
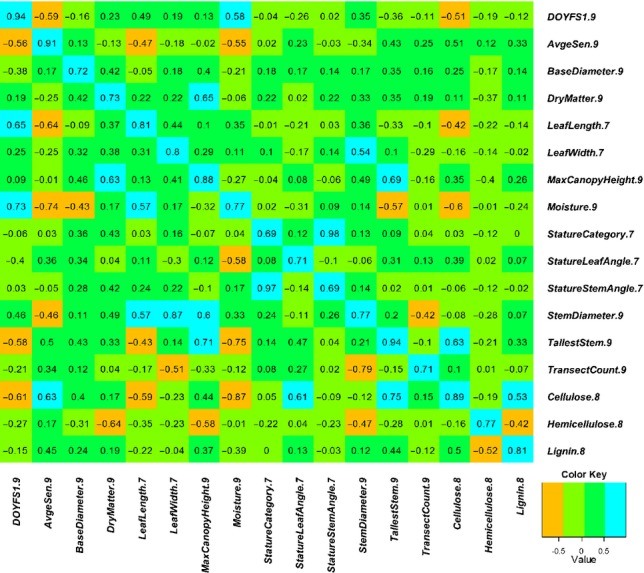
Genetic (below diagonal) and phenotypic (above diagonal) correlations among 17 phenotypic traits measured in 138 *Miscanthus sinensis* genotypes. Values on the main diagonal correspond to 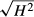, where *H*^2^ is the broad-sense heritability of the 17 traits (Table 4).

We defined an experimental population of 145 *M. sinensis* Anders. genotypes based on previous analyses of single-nucleotide polymorphism (SNP) data (Slavov *et al.*, 2013b). Of these, we attempted RAD sequencing for 142 and obtained robust data for 138 genotypes (see section on Sample clustering, population structure and relatedness), which were used for all subsequent analyses (Fig.1). In 2005, our study population was planted at 1.5 × 1.5 m spacing in a replicated field trial located near Aberystwyth (Wales, UK), following a randomized complete block design, with one replicate per genotype in each of four blocks. The field trial has been described in greater detail in several previous studies (Allison *et al.*, 2011; Jensen *et al.*, 2011; Robson *et al.*, 2012). Phenotyping and DNA extraction protocols have been described by Slavov *et al.* (2013b). Briefly, 17 phenotypic traits reflecting (1) phenology, (2) morphology and biomass productivity, and (3) cell wall composition, were measured on plants in all four replicates of the trial 2–4 yr after establishment (Table 1, Fig.2).

**Table 1:** Phenotypic traits measured in 142 *Miscanthus sinensis* genotypes

Trait^a^	Definition	*Q*_ST_^b^	*Geo*(*r*)^c^	PC1 (*P* value)^d^	PC2 (*P* value)^e^
Phenology					
*DOYFS1.9*	Date of flowering stage 1: day of year when the first flag leaf emerged	**0.24**	**Alt (−0.66)**	**0.42 (< 0.001)**	**0.19 (0.039)**
*AvgeSen.9*	Average senescence score (0–10) throughout the growing season	0.04	**Lat (0.46)**	**−0.19 (0.024)**	0.03 (0.752)
Morphology/biomass					
*BaseDiameter.9*	Largest plant diameter measured at ground level (mm)	0.03	Long (0.18)	**−**0.09 (0.285)	**−0.30 (< 0.001)**
*DryMatter.9*	Estimated total dry weight (g)	0.01	Alt (**−**0.20)	**−**0.09 (0.295)	**−**0.06 (0.497)
*LeafLength.7*	Ligule-to-tip length along the central vein of the youngest leaf with a ligule (cm)	**0.34**	**Alt (−0.59)**	**0.46 (< 0.001)**	**0.21 (0.013)**
*LeafWidth.7*	Blade width at half-leaf length for the leaf used to measure *LeafLength* (cm)	0.00	Lat (**−**0.21)	0.09 (0.270)	**−0.27 (0.001)**
*MaxCanopyHeight.9*	Height from the ground to the point of ‘inflection’ of the majority of leaves (cm)	0.18	Lat (**−**0.23)	**−0.29 (0.001)**	**−**0.03 (0.729)
*Moisture.9*	Estimated moisture content based on a subsample (%)	**0.34**	**Alt (−0.66)**	**0.49 (< 0.001)**	0.01 (0.946)
*StatureCategory.7*	Additive combination of *StatureLeafAngle* and *StatureStemAngle*	0.02	**Long (0.37)**	**−0.19 (0.030)**	**−**0.16 (0.054)
*StatureLeafAngle.7*	Three-category score reflecting leaf angle relative to the vertical	0.06	Alt (0.19)	**−**0.16 (0.059)	**0.18 (0.035)**
*StatureStemAngle.7*	Four-category score reflecting stem angle relative to the vertical	0.00	**Long (0.36)**	**−**0.15 (0.081)	**−0.20 (0.017)**
*StemDiameter.9*	Diameter 10–15 cm from the ground of a randomly chosen stem (mm)	0.00	**Alt (−0.25)**	0.08 (0.375)	**−**0.08 (0.332)
*TallestStem.9*	Length of the tallest stem (cm)	**0.46**	**Alt (0.57)**	**−0.53 (< 0.001)**	**−**0.08 (0.361)
*TransectCount.9*	Number of stems with ≥ 50% canopy height across the middle of the plant	0.00	Lat (0.19)	0.08 (0.370)	0.02 (0.787)
Cell wall composition					
*Cellulose.8*	Gravimetrically measured cellulose content (% dry weight)	**0.23**	**Alt (0.54)**	**−0.41 (< 0.001)**	0.02 (0.793)
*Hemicellulose.8*	Gravimetrically measured hemicellulose content (% dry weight)	0.06	Long (0.22)	**−0.21 (0.015)**	0.13 (0.117)
*Lignin.8*	Gravimetrically measured lignin content (% dry weight)	0.00	Long (**−**0.17)	0.00 (0.959)	0.03 (0.770)

a Trait, phenotypic traits measured in 2007 (.7), 2008 (.8) or 2009 (.9) (i.e. after two, three or four growing seasons, respectively). Detailed phenotyping protocols have been described by Slavov *et al.* (2013b).

b *Q*_ST_, genetic differentiation between ‘Continent’ and ‘Japan’ subpopulations. Values exceeding the empirical 95th percentile of putatively neutral differentiation (i.e. *F*_ST_ = 0.23) are shown in bold (Slavov *et al.*, 2013b).

c *Geo*(*r*), geographical coordinate with strongest correlation and Pearson's correlation coefficient (Slavov *et al.*, 2013b). Values with two-sided *P *<* *0.05 are shown in bold.

d PC1 (*P* value), Pearson's correlation with the first eigenvector of population structure (Fig.1) and two-sided *P* value. Values with two-sided *P *<* *0.05 are shown in bold.

e PC2 (*P* value), Pearson's correlation with the second eigenvector of population structure (Fig.1) and two-sided *P* value. Values with two-sided *P *<* *0.05 are shown in bold.

### Phenotypic data analysis

Phenotypic data analyses have been described by Slavov *et al.* (2013b). Briefly, mixed linear models were used to estimate variance components and best linear unbiased predictors (BLUPs) for each trait. We also calculated phenotypic (Pearson's *r*) and genetic correlations among all pairs of traits (Fig.2). Genetic correlations were calculated using two different approaches. For pairs of traits measured in the same year, we calculated genetic correlations from estimates of genetic covariance (i.e. using Eqn 9, as described by Howe *et al.*, 2000). Alternatively, for pairs of traits measured in different years, we calculated genetic correlations from phenotypic correlations and estimates of broad sense heritability for each trait (i.e. Eqn 8, as described by Howe *et al.*, 2000).

### RAD sequencing

RAD library preparation was performed by Floragenex, Inc. (Eugene, OR, USA). Briefly, genomic DNA from each genotype was digested with the restriction endonuclease *Pst*I and processed into multiplexed RAD libraries following the methods described in previous studies (Baird *et al.*, 2008; Stölting *et al.*, 2013). *Pst*I adapters, each containing a unique 6-bp multiplex sequence index (barcode), were affixed to digested templates, polished and amplified via a polymerase chain reaction. The resulting RAD libraries were run on two Illumina (San Diego, CA, USA) HiSeq platforms at Ambry Genetics (Aliso Viejo, CA, USA) and the Oregon State University Center for Genome Research and Biocomputing (Corvallis, OR, USA) using Illumina 1 × 100-bp chemistry.

### RAD-Seq data analysis

Sequencing adaptors and barcodes were removed using Floragenex software tools, resulting in 94-bp RAD fragments. These trimmed fragments were then aligned to a reference genome sequence using Bowtie (Langmead *et al.*, 2009), and variants were called using SAMtools (Li *et al.*, 2009). The alignment and variant calling steps were repeated twice, using different reference sequences. First, we aligned all 94-bp RAD sequences from each genotype to v 1.0 of the *Sorghum bicolor* genome (Paterson *et al.*, 2009) and parsed the resulting SAMtools pileup files using custom scripts. In a second analysis, we created a skeleton *Miscanthus* pseudo-reference of RAD sequence data (Supporting Information Methods S1). This assembly contained 48 426 clustered RAD sequences, representing *c*. 4.1 Mb of *Miscanthus* genomic sequence. To further reduce the potential of identifying spurious SNVs from low-quality sequences, reads were trimmed from the 3′ end to a total length of 85 bp. SNV detection then proceeded as described previously. These two approaches resulted in the initial identification of 142 539 and 223 046 SNVs, respectively.

We used custom scripts and VCFtools (Danecek *et al.*, 2011) to further filter SNV data based on alignment statistics, minor allele frequency (MAF) and conformity of genotype frequencies to Hardy–Weinberg expectations (Table 2). For initial data analyses aimed at validating data quality and detecting patterns of population genetic structure, we generated sets of SNVs that passed relatively stringent filtering criteria, but without restricting MAFs (i.e. ‘stringent’ filtering, Table 2). To enhance the power of GWAS analyses and to assess the accuracy of genome-wide prediction as a function of the number of markers used, we also generated larger sets of SNVs using ‘liberal’ filtering criteria (Table 2). We used the PLINK software tool (Purcell *et al.*, 2007) to calculate the number of unlinked SNVs (*plink … –indep-pairwise 50 5 0.2*), as well as to estimate linkage disequilibrium (LD) (*plink … –ld-window-r2*).

**Table 2:** Filtering criteria for single-nucleotide variant (SNV) data from 138 *Miscanthus sinensis* genotypes

Filtering criteria	Stringent	Liberal
*Q*^a^	15	NA
*Min depth*^b^	14	3
*Min ave depth*^c^	NA	6
*Missing* (%)^d^	10	20
*Minor alleles*^e^	NA	3
*Max* |*F*_IS_|^f^	0.25	NA
*Min het reads*^g^	0.05	NA
*No. of alleles*^h^	2	2
Statistics		
*No. of loci* (*Sorg*)^i^	20 127	53 174
*Ave MAF* (*Sorg*)^j^	0.027	0.174
*Ave r*^2^_*1 kb*_ (*Sorg*)^k^	0.24	0.26
*No. of loci* (*Misc*)^l^	30 755	121 771
*Ave MAF* (*Misc*)^m^	0.031	0.115

a *Q*, minimum Phred-like SNV quality score (Li *et al.*, 2008).

b *Min depth*, minimum number of reads.

c *Min ave depth*, minimum average number of reads across all genotypes.

d *Missing* (%), maximum percentage of missing genotype data allowed for a given locus.

e *Minor alleles*, minimum number of copies of the minor allele among all genotypes.

f *Max* |*F*_IS_|, maximum deviation of observed genotype frequencies from Hardy–Weinberg expectations, *F*_*IS*_ = 1 – *H*_o_/*H*_e_, where *H*_o_ and *H*_e_ are the observed and expected heterozygosities.

g *Min het reads*, minimum proportion of reads supporting the less frequent allele in a heterozygous genotype.

h *No. of alleles*, number of SNV alleles detected.

i *No. of loci* (*Sorg*), number of SNVs that passed all filtering criteria based on alignments to the *Sorghum bicolor* genome.

j *Ave MAF* (*Sorg*), average minor allele frequency for SNVs that passed all filtering criteria based on alignments to the *Sorghum bicolor* genome.

k *Ave r*^2^_*1 kb*_ (*Sorg*), average linkage disequilibrium (*r*^2^, calculated as genotypic correlation) for pairs of loci with MAF ≥ 0.10 that aligned within 1 kb of one another in the *Sorghum bicolor* genome.

l *No. of loci* (*Misc*), number of SNVs that passed all filtering criteria based on alignments to an *M. sinensis* pseudo-reference.

m *Ave MAF* (*Misc*), average minor allele frequency for SNVs that passed all filtering criteria based on alignments to an *M. sinensis* pseudo-reference.

NA, not applicable.

### Sample clustering, population structure and relatedness

We used the individual-based principal component analysis (PCA) approach of Patterson *et al*. (2006) to detect outliers and to characterize population genetic structure based on SNV data filtered using our ‘stringent’ criteria (Table 2), after eliminating one marker from each pair of loci linked at *r*^2^ ≥ 0.8 (i.e. based on genotypic correlation). Four of the 142 sequenced genotypes were identified as outliers along four different and highly significant axes of variation (*P *< 10^−5^ from tests based on the Tracy–Widom distribution) based on the default settings of the *smartpca* program within the EIGENSOFT package (Patterson *et al.*, 2006). These genotypes had not been identified as outliers or potential *M. sacchariflorus* hybrids in analyses based on microsatellite and SNP data (Slavov *et al.*, 2013b), and we tentatively assumed that their detection as outliers based on RAD-Seq SNVs was an indication of inferior RAD-Seq data quality. These genotypes were therefore eliminated, and all subsequent analyses were performed using phenotypic and SNV data for the remaining 138 genotypes. However, by performing a subset of analyses based on all 142 genotypes, we also ensured that none of our major results depended on this decision.

Patterns of population structure detected using the PCA approach were also confirmed using the model-based clustering approach implemented in STRUCTURE (Pritchard *et al.*, 2000; Falush *et al.*, 2003, 2007), following the procedure used in a previous study (Slavov *et al.*, 2013b). Briefly, we ran STRUCTURE using the default model parameters and varying the assumed number of genetic groups (*K*) from one to six. Each run consisted of 10 000 burn-in iterations and 10 000 data collection iterations. We used the DISTRUCT program (Rosenberg, 2004) to visualize the results from 10 independent runs that had been aligned using the CLUMPP program (Jakobsson & Rosenberg, 2007). We also used the results from these runs to calculate the *ad hoc* statistic Δ*K*, which tends to peak at the value of *K* that corresponds to the highest hierarchical level of substructure (Evanno *et al.*, 2005), using the online version of the STRUCTURE HARVESTER program (Earl & von Holdt, 2012). Finally, we used the GCTA program (Yang *et al.*, 2011) to calculate the genetic relationship matrix (Yang *et al.*, 2010) among the 138 genotypes used in all analyses.

### GWASs

We used the efficient mixed-model association expedited approach (EMMAX; Kang *et al.*, 2010) to perform GWASs based on SNVs filtered using ‘liberal’ criteria (Table 2). To control for the confounding effects of cryptic relatedness and population structure, we incorporated the identity-by-state (IBS) kinship matrix (calculated on the basis of all markers using EMMAX) and the first two principal components of population structure (see Sample clustering, population structure and relatedness). This approach is widely used and is believed to provide adequate protection against both environmental and genetic confounding (Price *et al.*, 2010; Vilhjálmsson & Nordborg, 2013). We used data perturbation simulations (Yu *et al.*, 2006) to estimate the statistical power of our EMMAX analyses, as well as to quantify the inflation of naïve estimates of the proportion of variance explained (PVE) by each detected association (Beavis, 1998; Allison *et al.*, 2002; Ingvarsson *et al.*, 2008). For each iteration of the simulations, we randomly chose an SNV from our data and assigned constant additive effects −*a*, 0 and *a* (Falconer, 1989) to genotypes containing 0, 1 and 2 copies, respectively, of an arbitrarily chosen allele (all SNVs used in this study were bi-allelic). These effects were then added to phenotypic trait BLUPs, and the resulting data were analyzed using EMMAX, as described above. This approach preserves the overall structure of the data, whilst also allowing us to estimate statistical power across a range of allele effect sizes, as well as to compare estimated with expected PVE values. The expected value of PVE was calculated as:
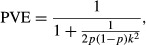
Eqn 1where *p* is the estimated frequency of an arbitrarily chosen SNV allele and *k* is the simulated additive effect (*a*) divided by the standard deviation of the respective trait BLUPs (we used *AvgeSen.9*). We chose values of *k* so that the expected PVE would be between 0.01 and 0.20 in 0.01 increments, and performed 1000 simulations for each of these values. Naïve estimates of PVE were calculated as the *R*^2^ from simple linear regression of the trait BLUP, including simulated additive effects, on SNV genotypes. This was only performed for SNVs that were significantly associated with the simulated trait BLUP at *α* = 10^−5^.

To assess the relative severity of confounding across traits, we also ran models that only included the IBS kinship matrix, or did not include any terms to account for population structure and relatedness (i.e. simple linear regression of trait BLUP on individual SNVs). In addition, we also used the multi-locus mixed-model (MLMM) approach of Segura *et al*. (2012) to perform a forward–backward model selection procedure and to potentially improve the power, whilst also reducing the rate of false positives of our GWAS analyses. We allowed up to nine forward selection and backward elimination steps and performed the procedure twice, based on MLMMs with or without the first two principal components of population structure included as fixed effects.

### Genome-wide prediction

We used the R package rrBLUP (Endelman, 2011) for genome-wide prediction using ridge regression. For a limited subset of analyses, we also used the Bayesian linear regression and the Bayesian LASSO approaches implemented in the BLR package (Perez *et al.*, 2010), but the performance of genome-wide prediction using these approaches was consistently comparable or lower than that using ridge regression, even after including the kinship matrix in the Bayesian models. To assess the effects of varying the size of the training population, we used 2–10-fold random cross-validations (i.e. random allocations of genotypes to training and test populations), which were repeated 100 times for each set of parameters.

Consistent with recommendations on standardizing analytical procedures and benchmarking (Daetwyler *et al.*, 2013), we quantified the performance of genome-wide prediction using three measures. First, we defined predictive ability (*r*) as Pearson's correlation of BLUPs calculated directly from field data and those obtained from the marker data using ridge regression (Daetwyler *et al.*, 2013). Second, we calculated prediction accuracies (*Accu*) by dividing predictive abilities by the square root of the broad-sense heritability (*H*) of the respective trait (Legarra *et al.*, 2008). Finally, we also recorded the intercepts (*b*_0_) and slopes (*b*_1_) of the simple linear regressions of BLUPs calculated from field data on those estimated using ridge regression. The last two measures, although not practically meaningful, can be indicative of model deficiencies and/or non-random partitioning of genotypes into training vs test populations (Daetwyler *et al.*, 2013).

To assess the potential of improving the performance of genome-wide prediction by selecting the most informative markers, we compared predictive abilities between cross-validations in which 10, 100, 1000 or 10 000 markers were chosen randomly, based on their GWAS significance (i.e. selecting loci with the lowest GWAS *P* values) within the training population, or based on their rrBLUP-estimated effects within the training population.

## Results

### RAD-Seq genotyping

**Fig 3:**
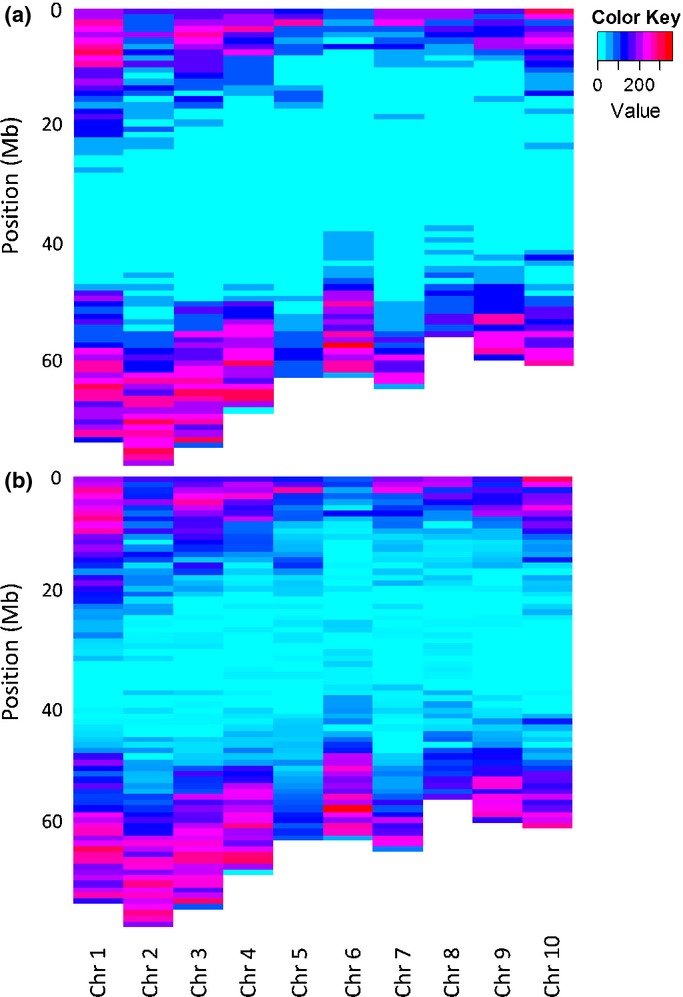
Chromosome-wide distribution of *Miscanthus sinensis* single-nucleotide variant loci detected based on alignments to *Sorghum bicolor* and filtered using ‘stringent’ (a) and ‘liberal’ (b) criteria (Table 2). Each line corresponds to a 1-Mb interval.

Our genotyping approach resulted in the detection of over 100 000 putative SNVs (see the Materials and Methods section). A large proportion of these SNVs did not satisfy even our ‘liberal’ filtering criteria, but even after ‘stringent’ filtering, over 20 000 informative loci were available for downstream analyses (Table 2). As expected, the chromosome-wide distribution of SNVs appeared to be biased, with very few SNVs detected in putative centromeric regions (Fig.3). At a finer scale, the vast majority of SNVs detected based on alignments to *Sorghum* (i.e. 98% and 96%, respectively, for ‘stringent’ and ‘liberal’ filtering) appeared to be located either inside or within 2 kb of genes.

As expected from the nature of RAD-Seq data, the majority of RAD-Seq SNVs were unlinked. For example, assuming microsynteny between *Sorghum* and *Miscanthus*, we used the sliding window LD-based pruning option of PLINK to calculate that 35 700 of the 53 174 SNVs detected based on alignments to *Sorghum* and ‘liberal’ filtering had no other SNV within a 25-SNV up- or downstream window that was linked at *r*^2^ > 0.2. Similarly, the 121 771 SNVs detected based on alignments to a *Miscanthus* pseudo-reference and ‘liberal’ filtering were located on 36 223 unique RAD tags. Although our data were not appropriate for detailed estimation of LD and historical rates of recombination (i.e. because of the largely unknown physical distances between SNVs in the *Miscanthus* genome), analyses based on SNVs located on the same RAD tag suggested that fine-scale LD is substantial (average *r*^2^ = 0.41 for SNVs with MAF ≥ 0.10, Fig. S1). Furthermore, analyses assuming microsynteny between *Sorghum* and *Miscanthus* clearly demonstrated that significant LD (average *r*^2^ ≥ 0.2) extends to at least several hundred bp (Table 2) and up to at least 1 Mb for some pairs of SNVs (Fig. S1).

### Population structure

Individual-based PCA based on SNVs filtered using ‘stringent’ criteria (Table 2) resulted in the identification of five significant eigenvectors (*P *< 10^−4^ from tests based on the Tracy–Widom distribution), which explained between 2.9% and 1.2% of the total SNV variation. As expected, PCA (Fig.1) and model-based clustering (Fig. S2) consistently delineated a ‘Continent’ vs ‘Japan’ genetic discontinuity, with both PC1 (Pearson's |*r*| = 0.83, *P *< 10^−15^) and PC2 (Pearson's |*r*| = 0.72, *P *< 10^−11^) being strongly correlated with the source longitudes of genotypes with known sampling locations. The differentiation between these two subpopulations was reflected in fine-scale patterns of LD, with *r*^2^ decaying slightly more slowly in each subpopulation than in the overall population (Fig. S1). Furthermore, the clear pattern of clustering based on PC1 and PC2, which accounted for 2.9% and 2.0% of the SNV variation, enabled us to form strong hypotheses about the geographical origins of 68% (47 of 69) of the accessions with unknown sampling locations (Fig.1).

### GWASs

As expected from the relatively small size of our population, data perturbation simulations indicated that GWAS analyses had very limited power to detect associations with small or moderate effects (PVE ≤ 0.10, Fig. S3). When such associations were detected, their estimated effects tended to be upwardly biased by as much as an order of magnitude (Fig. S3).

Naïve GWAS analyses that ignored the effects of population structure and relatedness (i.e. simple linear regression of trait BLUPs on individual SNVs) consistently resulted in severely inflated *P* values (i.e. quantile–quantile (QQ) plots in Figs S4, S5). This effect was particularly strong for traits characterized by strong genetic differentiation (i.e. high *Q*_ST_ between ‘Continent’ and ‘Japan’ subpopulations) and/or significant correlation with geographical variables or primary eigenvectors of population structure (Table 1, Figs S4, S5). The inclusion of the IBS kinship matrix substantially mitigated the confounding for all traits, whereas adding PC1 and PC2 further reduced the inflation of *P* values for some traits (i.e. *BaseDiameter.9*,*LeafWidth.7*,*TransectCount.9*), without apparently compromising statistical power relative to models that only included the IBS matrix (Figs S4, S5).

Using this conservative approach (i.e. including both the IBS kinship matrix and PC1 and PC2 as covariates in EMMAX analyses), we detected 35 putative associations (*P *< 10^−5^) for SNVs resulting from alignments to the *S. bicolor* genome (Fig. S4). Four of these associations (two for *AvgeSen.9,* one for *LeafLength.7* and one for *Lignin.8*) reached genome-wide significance after Bonferroni correction for multiple testing (*P *<* *0.05/53 174 ≈ 9.4 × 10^−7^), whereas another 13 had an estimated false discovery rate < 0.05 (Table 3). We detected similar patterns using SNVs resulting from alignments to an *M. sinensis* pseudo-reference (Fig. S5), with 53 SNVs reaching suggestive (*P *< 10^−5^) and two SNVs (one for *DOYFS1.9* and one for *TallestStem.9*) reaching Bonferroni-corrected genome-wide significance (*P *<* *0.05/121 771 ≈ 4.1 × 10^−7^).

**Table 3:** Markers with significant phenotypic associations (false discovery rate < 0.05) in 138 *Miscanthus sinensis* genotypes

Chromosome^a^	Position^b^	*P*^c^	*Q*^d^	MAF^e^	PVE^f^	Trait^g^	Gene^h^	Description/annotation
1	3 776 666	3.31E-06	0.03	0.01	0.12	*TallestStem.9*	Sb01g004700	ATVAMP725
1	3 789 996	2.98E-06	0.03	0.02	0.13	*TallestStem.9*	Sb01g004720	Aminoacyl-tRNA synthetase family
1	68 013 320	3.38E-06	0.03	0.01	0.12	*TallestStem.9*	Sb01g044850	Unknown protein
2	67 477 249	1.14E-06	0.03	0.01	0.16	*StatureStemAngle.7*	Sb02g032850	Unknown protein
2	67 477 259	1.14E-06	0.03	0.01	0.16	*StatureStemAngle.7*	Sb02g032850	Unknown protein
**3**	**8** **793** **225**	**6.38E-07**^i^^**,**^^j^^**,**^^k^	**0.03**	**0.49**	**0.18**	***LeafLength.7***	**Sb03g008300**	**DNA binding/protein dimerization**
**3**	**65** **293** **238**	**2.79E-06**[Table-fn tf3-12]	**0.03**	**0.05**	**0.02**	***AvgeSen.9***	**Sb03g037310**	**ATCDPMEK**
3	65 293 239	2.79E-06	0.03	0.05	0.02	*AvgeSen.9*	Sb03g037310	ATCDPMEK
4	4 150 586	1.57E-06	0.04	0.06	0.18	*LeafLength.7*	NA	NA
6	50 249 485	3.35E-06	0.03	0.02	0.16	*TallestStem.9*	Sb06g020830	Protein kinase family protein
**6**	**54** **211** **630**	**8.64E-07**^i^^**,**^^j^^**,**^^k^	**0.05**	**0.17**	**0.15**	***Lignin.8***	**Sb06g025250**	**Serine-type endopeptidase/serine-type peptidase**
**6**	**58** **247** **799**	**5.66E-07**^i^^**,**^^j^	**0.02**	**0.03**	**0.25**	***AvgeSen.9***	**Sb06g029670**	**ATP binding/protein kinase/protein serine/threonine kinase/protein tyrosine kinase/sugar binding**
9	24 591 741	1.84E-06	0.03	0.03	0.26	*AvgeSen.9*	NA	NA
9	46 480 666	4.07E-06	0.04	0.15	0.02	*AvgeSen.9*	Sb09g018620	Hydroxyproline-rich glycoprotein family protein
**10**	**49** **962** **793**	**4.90E-07**^i^^**,**^^k^	**0.02**	**0.04**	**0.21**	***AvgeSen.9***	**Sb10g022360**	**Unknown protein**
**10**	**55** **336** **585**	**3.14E-06**^j^	**0.03**	**0.01**	**0.12**	***TallestStem.9***	**Sb10g026010**	**UBP19; cysteine-type endopeptidase/ubiquitin thiolesterase**
**10**	**59** **574** **096**	**1.50E-06**^j^	**0.03**	**0.05**	**0.19**	***TallestStem.9***	**Sb10g029835**	**Unknown protein**

Associations with Bonferroni-corrected genome-wide significance (*α* = 0.05) are shown in bold. Only results for markers detected from alignments to the *Sorghum bicolor* genome are shown.

a Chromosome, *Sorghum bicolor* chromosome to which the marker was aligned.

b Position, *Sorghum bicolor* chromosome position to which the marker was aligned.

c *P*,*P* value from genome-wide association studies (GWAS) analysis using the efficient mixed-model association expedited approach (EMMAX), including the kinship matrix and the first two eigenvectors of population structure (see the Materials and Methods section).

d *Q*, false discovery rate calculated using the *q*-value R package (Dabney & Storey, 2013).

e MAF, minor allele frequency.

f PVE, naïve estimate of the proportion of variance explained based on simple linear regression (see the Materials and Methods section).

g Trait, phenotypic trait as defined in Table 1.

h Gene, *Sorghum bicolor* gene to which the marker was aligned.

i Significant at genome-wide *α* = 0.05 after Bonferroni correction based on EMMAX analyses (see the Materials and Methods section).

j Included in the optimal model according to the multiple Bonferroni criterion in multi-locus mixed-model (MLMM) analyses (see the Materials and Methods section).

k Included in the optimal model according to the multiple Bonferroni criterion in MLMM analyses including the first two eigenvectors of population structure (see the Materials and Methods section).

NA, not applicable (markers aligning to putatively intergenic positions).

Results from the MLMM-GWAS approach were generally consistent with those from the single-locus EMMAX analyses (Figs S4, S6). However, the MLMM approach allowed us to detect Bonferroni-corrected associations for two additional traits (*BaseDiameter.9* and *TallestStem.9*). Furthermore, when PC1 and PC2 were included as covariates in MLMMs, the selection procedure for *AvgeSen.9* favored a model including six significantly associated SNVs (Fig. S7).

### Genome-wide prediction

**Fig 4:**
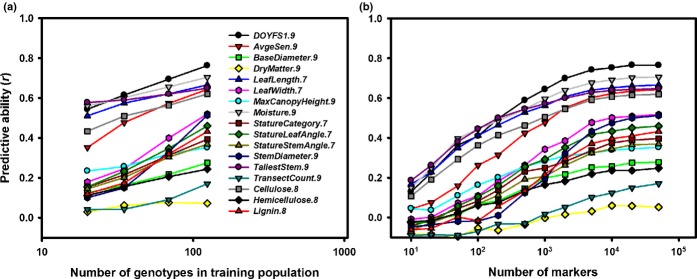
Performance of genome-wide prediction in a population of 138 *Miscanthus sinensis* genotypes using single-nucleotide variant markers obtained from alignments to the *Sorghum bicolor* genome. Predictive ability as a function of training population size (a) and number of markers used (b). All data points are averages across 100 random cross-validations.

**Fig 5:**
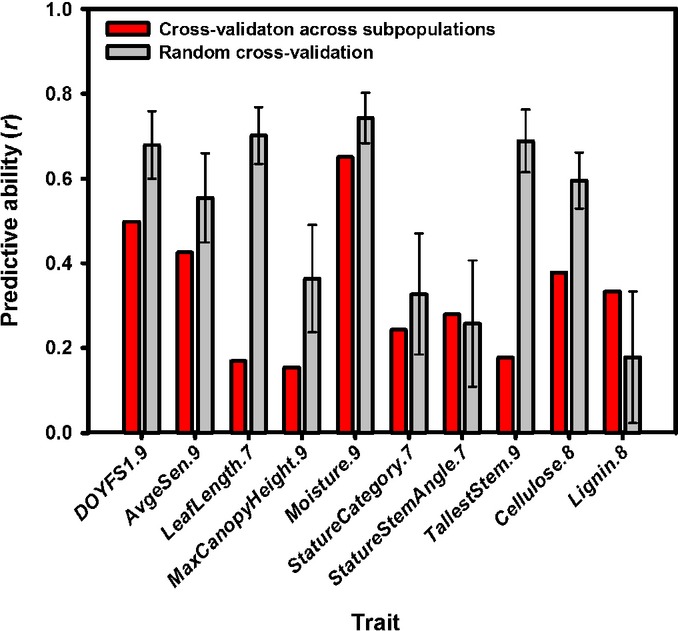
Effect of population structure on the performance of genome-wide prediction in a population of 70 *Miscanthus sinensis* genotypes with known sampling locations using single-nucleotide variants obtained from alignments to the *Sorghum bicolor* genome. Cross-validations across subpopulations (red bars) were performed using genotypes from Japan (*N *=* *43) as a training population and genotypes from China and South Korea (*N *=* *27) as a test population. Random cross-validations (gray bars) were performed by randomly selecting the same numbers of genotypes in the training and test populations from the total set of 138 genotypes. All data points are averages, and error bars correspond to standard deviations across 100 random cross-validations. Only traits with cross-subpopulation predictive abilities exceeding (1) 0.10 and (2) the standard deviation from the random cross-validations for the respective trait are shown.

**Fig 6:**
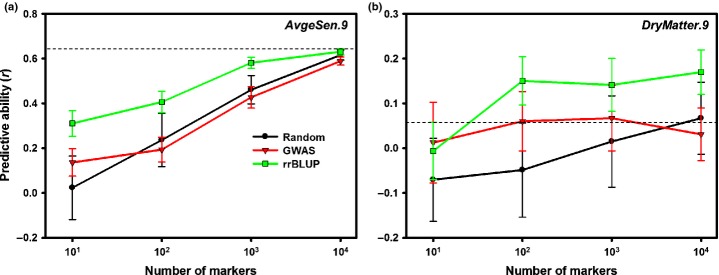
Effect of marker selection on the performance of genome-wide prediction of average senescence score (a) and total dry weight (b) in a population of 138 *Miscanthus sinensis* genotypes based on single-nucleotide variants (SNVs) detected from alignments to the *Sorghum bicolor* genome. ‘Random’ (circles, black line), randomly selected markers; ‘GWAS’ (triangles, red line), markers with the lowest genome-wide association study *P* values within the training population; ‘rrBLUP’ (squares, green line), markers with the highest estimated effects within the training population based on ridge regression. All data points are averages ± SD across 100 random cross-validations. Dashed lines correspond to predictive abilities based on all 53 174 SNVs.

As expected, predictive abilities were moderately correlated with broad-sense heritabilities (Pearson's *r *>* *0.57, *P *<* *0.018, Table 4) and appeared to increase monotonically with the size of the training population (Fig.4a). Although considerable variation was present among the 17 traits, the intercepts and slopes of simple linear regressions of BLUPs calculated from field data on those estimated using ridge regression tended to be close to their expected values of 0.00 and 1.00, respectively (Table S1). Interestingly, both predictive abilities and accuracies of genome-wide prediction were statistically indistinguishable (*P *>* *0.64 from paired *t*-tests across traits) between the sets of SNVs obtained based on different alignments (Table 4). Furthermore, predictive abilities tended to reach a plateau when *c*. 10 000–20 000 markers were used (i.e. approximately half of the *c*. 35 000–36 000 presumably independent loci), with further increases resulting in little or no improvement (Fig.4b). Genetic structure (Fig.1) and the presence of closely related genotypes in the training and test populations (Fig. S8) affected the performance of genome-wide prediction, with predictive abilities from cross-validations across subpopulations being significantly lower than those from corresponding random cross-validations (one-sided *P *=* *0.0004 from a paired *t*-test across traits). However, the extent of this difference varied considerably among traits (Fig.5). Finally, the selection of markers based on their GWAS significance or rrBLUP-estimated effects appears to have potential for improving predictive abilities (Fig.6), particularly for traits with lower heritabilities (Fig.6b).

**Table 4:** Performance of genome-wide prediction in 138 *Miscanthus sinensis* genotypes based on single-nucleotide variant (SNV) markers filtered using liberal criteria (Table 2)

Trait^a^	*H* ^2^ b	*r*_*Sorg*_ (SD)^c^	*Accu*_*Sorg*_ (SD)^d^	*r*_*Misc*_ (SD)^e^	*Accu*_*Misc*_ (SD)^f^
Phenology					
*DOYFS1.9*	0.89	0.76 (0.02)	0.81 (0.02)	0.78 (0.01)	0.82 (0.02)
*AvgeSen.9*	0.83	0.64 (0.01)	0.71 (0.01)	0.64 (0.01)	0.71 (0.01)
Morphology/biomass					
*BaseDiameter.9*	0.52	0.27 (0.05)	0.38 (0.06)	0.29 (0.04)	0.40 (0.06)
*DryMatter.9*	0.54	0.06 (0.05)	0.09 (0.07)	0.04 (0.06)	0.05 (0.08)
*LeafLength.7*	0.65	0.67 (0.01)	0.83 (0.01)	0.66 (0.01)	0.82 (0.01)
*LeafWidth.7*	0.64	0.52 (0.02)	0.65 (0.03)	0.56 (0.01)	0.70 (0.02)
*MaxCanopyHeight.9*	0.77	0.35 (0.03)	0.40 (0.03)	0.34 (0.02)	0.39 (0.03)
*Moisture.9*	0.59	0.70 (0.01)	0.92 (0.01)	0.73 (0.01)	0.95 (0.01)
*StatureCategory.7*	0.48	0.39 (0.03)	0.57 (0.04)	0.43 (0.02)	0.62 (0.03)
*StatureLeafAngle.7*	0.50	0.46 (0.03)	0.65 (0.05)	0.47 (0.02)	0.66 (0.03)
*StatureStemAngle.7*	0.48	0.37 (0.02)	0.53 (0.03)	0.40 (0.02)	0.58 (0.03)
*StemDiameter.9*	0.60	0.51 (0.03)	0.66 (0.04)	0.50 (0.02)	0.65 (0.03)
*TallestStem.9*	0.88	0.65 (0.01)	0.69 (0.01)	0.63 (0.01)	0.68 (0.02)
*TransectCount.9*	0.51	0.17 (0.04)	0.23 (0.06)	0.27 (0.03)	0.39 (0.04)
Cell wall composition					
*Cellulose.8*	0.79	0.62 (0.02)	0.70 (0.02)	0.61 (0.02)	0.69 (0.02)
*Hemicellulose.8*	0.60	0.25 (0.03)	0.32 (0.04)	0.18 (0.04)	0.24 (0.05)
*Lignin.8*	0.66	0.43 (0.02)	0.53 (0.03)	0.35 (0.02)	0.43 (0.03)
Average (SD)^g^	0.64	0.46 (0.20)	0.57 (0.22)	0.46 (0.20)	0.57 (0.23)

All predictive abilities and accuracies are based on 100 random 10-fold cross-validations (i.e. using a training population with *N *=* *124 genotypes).

a Trait, phenotypic trait as defined in Table 1.

b *H*^2^, broad-sense heritability (see the Materials and Methods section).

c *r*_*Sorg*_ (SD), average predictive ability and standard deviation across 100 random 10-fold cross-validations based on 53 174 SNVs obtained from alignments to the *Sorghum bicolor* genome.

d *Accu*_*Sorg*_ (SD), average accuracy of genome-wide prediction and standard deviation across 100 random 10-fold cross-validations based on 53 174 SNVs obtained from alignments to the *S. bicolor* genome

e *r*_*Misc*_ (SD), average predictive ability and standard deviation across 100 random 10-fold cross-validations based on 121 771 SNVs obtained from alignments to an *M. sinensis* pseudo-reference.

f *Accu*_*Misc*_ (SD), average accuracy of genome-wide prediction and standard deviation across 100 random 10-fold cross-validations based on 121 771 SNVs obtained from alignments to an *M. sinensis* pseudo-reference.

g Average (SD), overall average and standard deviation across traits.

## Discussion

As in a number of previous studies across a wide phylogenetic range of plant species (Barchi *et al.*, 2011; Chutimanitsakun *et al.*, 2011; Stölting *et al.*, 2013), the application of RAD-Seq genotyping resulted in the generation of large numbers of informative SNV markers (Table 2). Alignments of RAD tags to an *M. sinensis* pseudo-reference resulted in the identification of substantially greater numbers of markers (Table 2) and indistinguishable performance of genome-wide prediction (Table 4) compared with alignments to the *S. bicolor* genome. However, it is possible that the availability of a reference genome sequence from a closer relative would have resulted in more informative RAD-Seq SNV data. In any case, our results indicate that the *a priori* availability of a high-quality reference genome sequence does not appear to be a requirement for the success of this genotyping procedure.

Although we were able to generate data for a large number of RAD-Seq markers, the use of a methylation-sensitive enzyme (*Pst*I) and relatively stringent alignment criteria resulted in a greatly unbalanced genome coverage (Fig.3) and strong bias against intergenic SNVs. Although probably advantageous in terms of data quality, this effect may limit the applicability of RAD-Seq genotyping for the complete dissection of complex trait architecture because over half of trait-associated polymorphisms may be located outside of genes (Li *et al.*, 2012). However, this shortcoming could possibly be mitigated by using multiple restriction enzymes with varying sensitivities to methylation.

The patterns of putatively neutral population structure detected based on RAD-Seq SNVs (Fig.1) were consistent with those described previously based on much smaller numbers of markers (Slavov *et al.*, 2013b). However, the greater power of the RAD-Seq markers allowed us to detect additional nuances, as well as to form hypotheses about the geographical origins of over two-thirds of the genotypes with unknown sampling locations (Fig.1). Extensions of this approach to wider germplasm collections (including other *Miscanthus* species) and more sophisticated models (Baran *et al.*, 2013) will significantly improve our knowledge about the evolutionary history of the genus and will provide important practical information to breeders.

Despite the small size of our association mapping population (*N *=* *138), seven associations consistently reached genome-wide significance across a range of GWAS analyses and adjustments for multiple testing (Table 3, Figs S4–S7). Interestingly, two of these associations (i.e. one for *AvgeSen.9* and one for *Lignin.8*; Table 3), as well as another two putative associations for *StemDiameter.9* and *TallestStem.9* (*P *< 10^−5^, Fig. S4), all appeared to align within 8–16 Mb of the putative dwarfing locus *dw2*, whose location was recently supported by a GWAS for plant height in *S. bicolor* (Morris *et al.*, 2013). This region may therefore require particular attention in future association and linkage mapping studies.

What is the explanation for the detection of multiple significant associations, given the relatively limited statistical power of our GWASs (Fig. S3)? Because no single unambiguous explanation can be provided, we hypothesize that several factors may underlie this observation. First, LD in *M. sinensis* (Fig. S1) does not appear to be as extensive as in its primarily self-pollinating relative *S. bicolor* (Morris *et al.*, 2013). However, there was clear evidence of long-range LD in at least some regions of the *M. sinensis* genome (Fig. S1). Thus, although the initial stages of follow-up studies will focus on the regions in the immediate vicinity of trait-associated SNVs, it is conceivable that at least some of these are tagging causative polymorphisms located many kilobases away. Second, the statistical power to detect associations of small to moderate effect (PVE ≤ 0.10) was only between 0.001 and 0.213 (Fig. S3). However, if the genomic architectures of the traits that we studied are highly complex, dozens or even hundreds of causative polymorphisms with minor effects may exist across the genome. Under this highly polygenic scenario, even an underpowered study that uses a large number of markers is likely to detect a subset of minor effect associations and dramatically overestimate their PVE (Fig. S3). Alternatively, some of the SNVs detected by our GWASs may be linked to causative polymorphisms of larger effects. However, the former scenario seems to be more plausible based on larger scale GWAS results in other related crops (Buckler *et al.*, 2009; Morris *et al.*, 2013). Furthermore, the highly polygenic scenario is consistent with the results from our assessment of marker selection strategies for genome-wide prediction (Fig.6), which was performed through cross-validation and should therefore be immune to the inflation of effect sizes. Finally, it is possible that our analytical procedures failed to control the rate of false positives. However, our results (i.e. QQ plots in Figs S4, S5) are generally inconsistent with this explanation.

Although GWAS results are expected to improve in the near future (i.e. with the use of larger populations and denser genome coverage), the ability to predict phenotypes from a genome-wide set of markers is likely to have immediate impact on *Miscanthus* breeding programs. Our genome-wide prediction results illustrate several important points. First, as expected from both theoretical and empirical studies (Daetwyler *et al.*, 2008, 2010, 2013; Resende *et al.*, 2012), trait heritability was correlated with predictive ability. However, this correlation was only moderate, confirming that other factors (e.g. genomic architecture of the trait, LD, effective population size) may be equally important (Daetwyler *et al.*, 2010; de Los Campos *et al.*, 2013). For example, *DryMatter.9* was moderately heritable (*H*^2^ = 0.54), yet genome-wide prediction of this trait consistently failed (Table 4, Fig.4). This is not surprising, given the highly composite nature of biomass yield, and targeting individual yield components or correlates (e.g. *MaxCanopyHeight.9* and *StemDiameter.9*; Fig.2) appears to be a more promising approach (Table 4, Fig.4). In contrast, the accuracy of genome-wide prediction for *Moisture.9* (*H*^2^ = 0.59) nearly reached its theoretical maximum (Table 4), suggesting that genomic selection for this trait may be feasible even with very small training populations (i.e. *N *<* *100, Fig.4a), with as few as 100–1000 markers (Fig.4b) and across subpopulations (Fig.5). Predictive ability was similarly high for phenological traits (i.e. *DOYFS1.9* and *AvgeSen.9*), as well as *Cellulose.8*, and promising for most other traits (i.e. except *DryMatter.9* and *TransectCount.9*; Table 4, Fig.4). We are therefore exploring the practical application and validation of genomic selection in the *Miscanthus* breeding program at the Institute of Biological, Environmental and Rural Sciences (IBERS). Second, our results clearly suggest that substantial further improvements in predictive ability are more likely to come from using larger training populations (Fig.4a) than from using denser genome coverage (Fig.4b). However, an important caveat is that the SNVs that we used probably did not cover vast regions of the *Miscanthus* genome (Fig.3). The observed plateau in predictive ability when 10 000–20 000 SNVs were used clearly needs to be validated using more representative samples of markers. However, based on our results and currently available genotyping platforms, our recommended approach to genome-wide prediction in non-model plants would be to maximize the number of individuals in the training population and to use a low-cost genotyping strategy (Davey *et al.*, 2011; Elshire *et al.*, 2011; Poland *et al.*, 2012). Third, the robustness of genome-wide prediction across subpopulations varied dramatically among traits (Fig.5). Thus, the relationship between training and test populations needs to be characterized in detail, and individuals from the populations targeted by genomic selection (or their close relatives) should ideally be included in training populations. Finally, our preliminary assessment of locus selection strategies (Fig.6) clearly indicated that there is great potential to increase predictive abilities through the application of sophisticated analytical approaches (de Los Campos *et al.*, 2013), which do not attempt to estimate phenotypic effects for all loci (i.e. in contrast to the method we used). The refinement of these approaches and their combination with multi-locus and multi-trait procedures (Korte *et al.*, 2012; Segura *et al.*, 2012) offer exciting prospects for the characterization of pleiotropy and the dissection of highly complex phenotypic traits. However, studies aimed at pushing the limits of genome-wide prediction cannot overcome the inherent limitations of this approach (e.g. trait heritability sets an upper limit on accuracy) and need to be designed and interpreted with awareness of its numerous pitfalls (Wray *et al.*, 2013).

In summary, we related high-quality phenotypic data for 17 traits in a population of *M. sinensis* to SNV markers obtained through RAD-Seq genotyping. Despite the relatively small size of our experimental population (*N *=* *138), results from GWASs were promising and suggest that this approach will be instrumental for the dissection of complex phenotypic traits. On a more immediate time-scale, results from our genome-wide prediction analyses suggest that the application of genomic selection in *Miscanthus* may be feasible, and we are therefore validating this finding in our accelerated breeding program.

## Supporting Information

Fig S1 Linkage disequilibrium based on single-nucleotide variants detected using restriction site-associated DNA sequencing in *Miscanthus sinensis*. **Fig. S2** Model-based clustering of single-nucleotide variant data for 138 *Miscanthus sinensis* genotypes. **Fig. S3** Statistical power and effect size inflation based on data perturbation simulations for genome-wide association study analyses using the EMMAX program. **Fig. S4** Genome-wide association study (GWAS) results for 17 phenotypic traits in a population of 138 *Miscanthus sinensis* genotypes based on 53 174 markers detected using alignments to the *Sorghum bicolor* genome. **Fig. S5** Genome-wide association study (GWAS) results for 17 phenotypic traits in a population of 138 *Miscanthus sinensis* genotypes based on 121 771 markers detected using alignments to a *M. sinensis* pseudo-reference. **Fig. S6** Multi-locus mixed-model (MLMM) genome-wide association study results for 17 phenotypic traits in a population of 138 *Miscanthus sinensis* genotypes based on 53 174 markers detected using alignments to the *Sorghum bicolor* genome. **Fig. S7** Covariate multi-locus mixed-model (MLMM) genome-wide association study (GWAS) results for average senescence score in a population of 138 *Miscanthus sinensis* genotypes based on 53 174 markers detected using alignments to the *Sorghum bicolor* genome. **Fig. S8** Genetic relationship matrix among 138 *Miscanthus sinensis* genotypes. **Table S1** Simple linear regression measures of performance of genome-wide prediction in a population of 138 *Miscanthus sinensis* genotypes **Methods S1***Miscanthus* pseudo-reference.
