# Supplementary material for: Genome-wide association studies and prediction of 17 traits related to phenology, biomass and cell wall composition in the energy grass Miscanthus sinensis
Source: New Phytol. 2013 Dec 6;201(4):1227–39. doi: 10.1111/nph.12621 (PMC4284002; doi:10.1111/nph.12621)
Supplement: Fig S1 — Linkage disequilibrium based on single-nucleotide variants detected using restriction site-associated DNA sequencing in Miscanthus sinensis. Fig. S2 Model-based clustering of single-nucleotide variant data for 138 Miscanthus sinensis genotypes. Fig. S3 Statistical power and effect size inflation based on data perturbation simulations for genome-wide association study analyses using the EMMAX program. Fig. S4 Genome-wide association study (GWAS) results for 17 phenotypic traits in a population of 138 Miscanthus sinensis genotypes based on 53 174 markers detected using alignments to the Sorghum bicolor genome. Fig. S5 Genome-wide association study (GWAS) results for 17 phenotypic traits in a population of 138 Miscanthus sinensis genotypes based on 121 771 markers detected using alignments to a M. sinensis pseudo-reference. Fig. S6 Multi-locus mixed-model (MLMM) genome-wide association study results for 17 phenotypic traits in a population of 138 Miscanthus sinensis genotypes based on 53 174 markers detected using alignments to the Sorghum bicolor genome. Fig. S7 Covariate multi-locus mixed-model (MLMM) genome-wide association study (GWAS) results for average senescence score in a population of 138 Miscanthus sinensis genotypes based on 53 174 markers detected using alignments to the Sorghum bicolor genome. Fig. S8 Genetic relationship matrix among 138 Miscanthus sinensis genotypes. Table S1 Simple linear regression measures of performance of genome-wide prediction in a population of 138 Miscanthus sinensis genotypes Methods S1Miscanthus pseudo-reference. [file nph0201-1227-SD1.pdf]

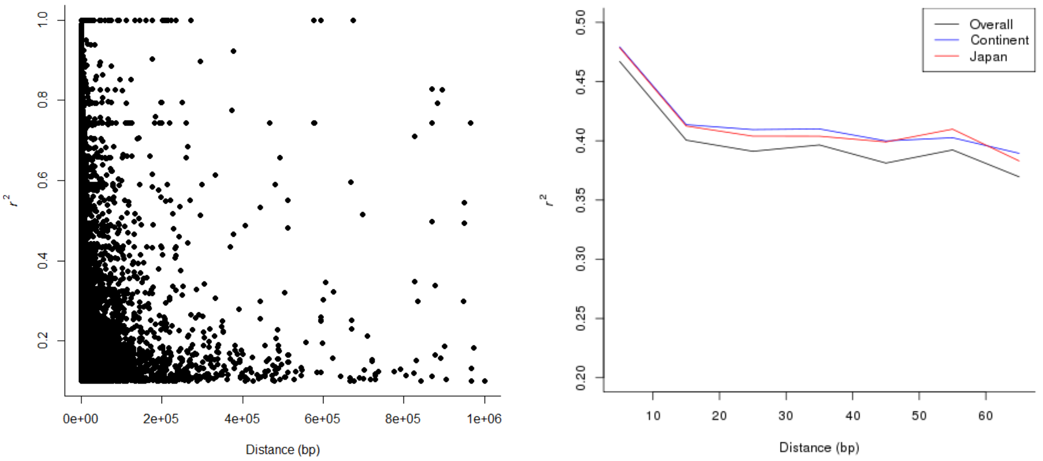

Figure S1 Linkage disequilibrium (LD, measured as pairwise r^2 from genotypic correlation) based on single-nucleotide variants (SNVs) detected using restriction-site associated DNA sequencing (RAD-Seq) in *Miscanthus sinensis*. Left: large-scale LD among SNVs that were (1) detected using alignments to the *Sorghum bicolor* genome; (2) filtered using 'liberal' criteria; and (3) located within 1 Mb of each other, assuming microsynteny between *Miscanthus* and *Sorghum*. Only values of $r^2 \geq 0.1$ are shown. Right: fine-scale LD among SNVs that (1) were detected using alignments to a *Miscanthus* pseudo-reference; (2) were filtered using 'liberal' criteria; (3) were located within the same RAD tag; and (4) had minor allele frequencies of at least 0.10. To equalize sample sizes, r^2 values were calculated based on N = 26 individuals from the overall population, as well as from the 'Continent' and 'Japan' subpopulations.

(**a**)

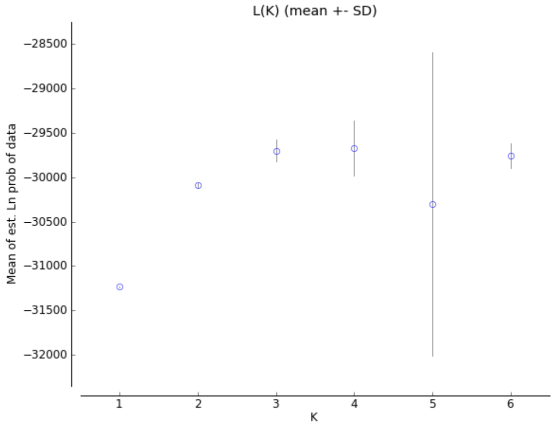

(**b**)

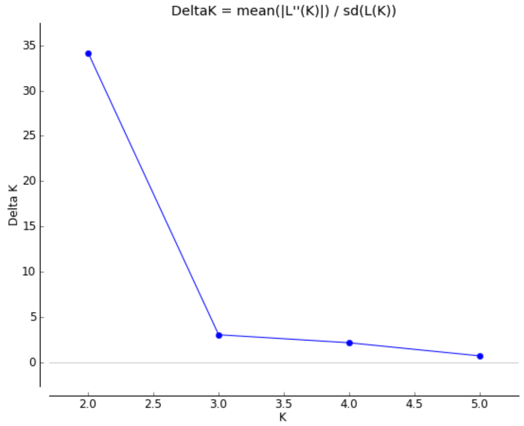

(**c**)

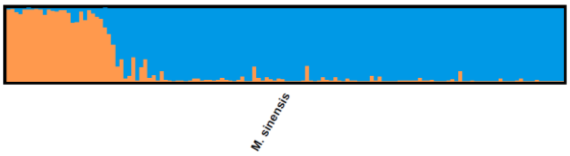

Figure S2 Model-based clustering of single-nucleotide variant data for 138 *M. sinensis* genotypes using v. 2.3.4 of the STRUCTURE program (Pritchard *et al.*, 2000; Falush *et al.*, 2003, 2007). (a) Mean log-likelihoods and their standard deviations from runs assuming different numbers of subpopulations (K). (b) Values of the *ad hoc* statistic ΔK, which tends to peak at the value of K that corresponds to the highest hierarchical level of substructure (Evanno *et al.*, 2005). (c) Individual proportional memberships of 138 *M. sinensis* genotypes assuming K = 2. Clustering results from 10 independent runs of STRUCTURE were aligned using the CLUMPP program (Jakobsson & Rosenberg, 2007) and illustrated using the DISTRUCT program (Rosenberg, 2004). Genotypes were ordered by their scores on the primary axis of variation (PC1) detected using individual-based principal component analysis (Fig. 1, Patterson *et al.*, 2006).

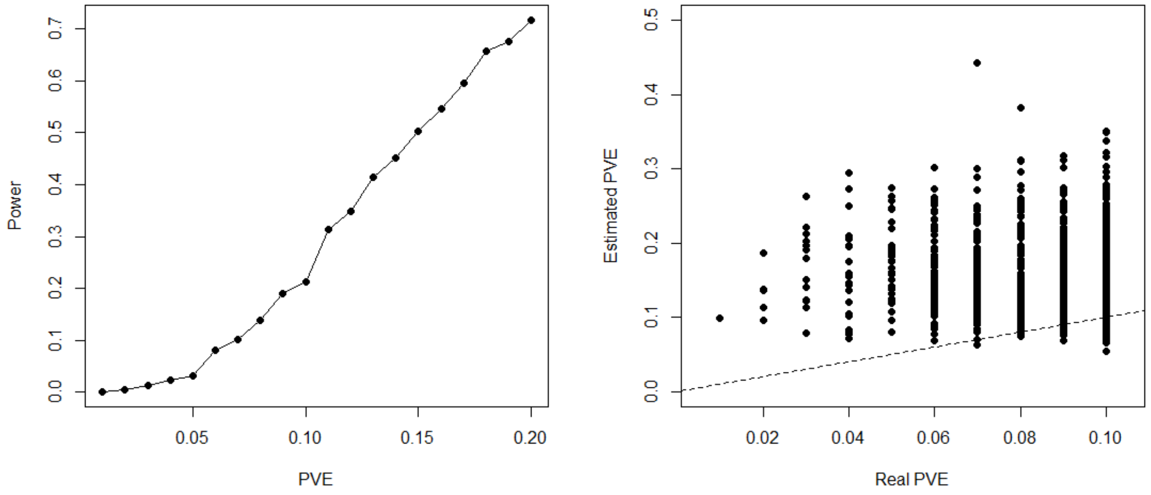

Figure S3 Statistical power (left) and effect size inflation (right) based on data perturbation simulations (Yu *et al.*, 2006) for genome-wide association study (GWAS) analyses using the EMMAX program, with mixed linear models including the identity-by-state matrix and the first two eigenvectors of population structure. Left: statistical power was estimated for $\alpha = 10^{-5}$ and setting a target proportion of variance explained (PVE, see Materials and Methods). Right: naïve estimates of (PVE) for associations detected at $\alpha = 10^{-5}$ vs their 'real' simulated values. In the absence of bias, points are expected to follow a trend around the x = y diagonal (dashed line).

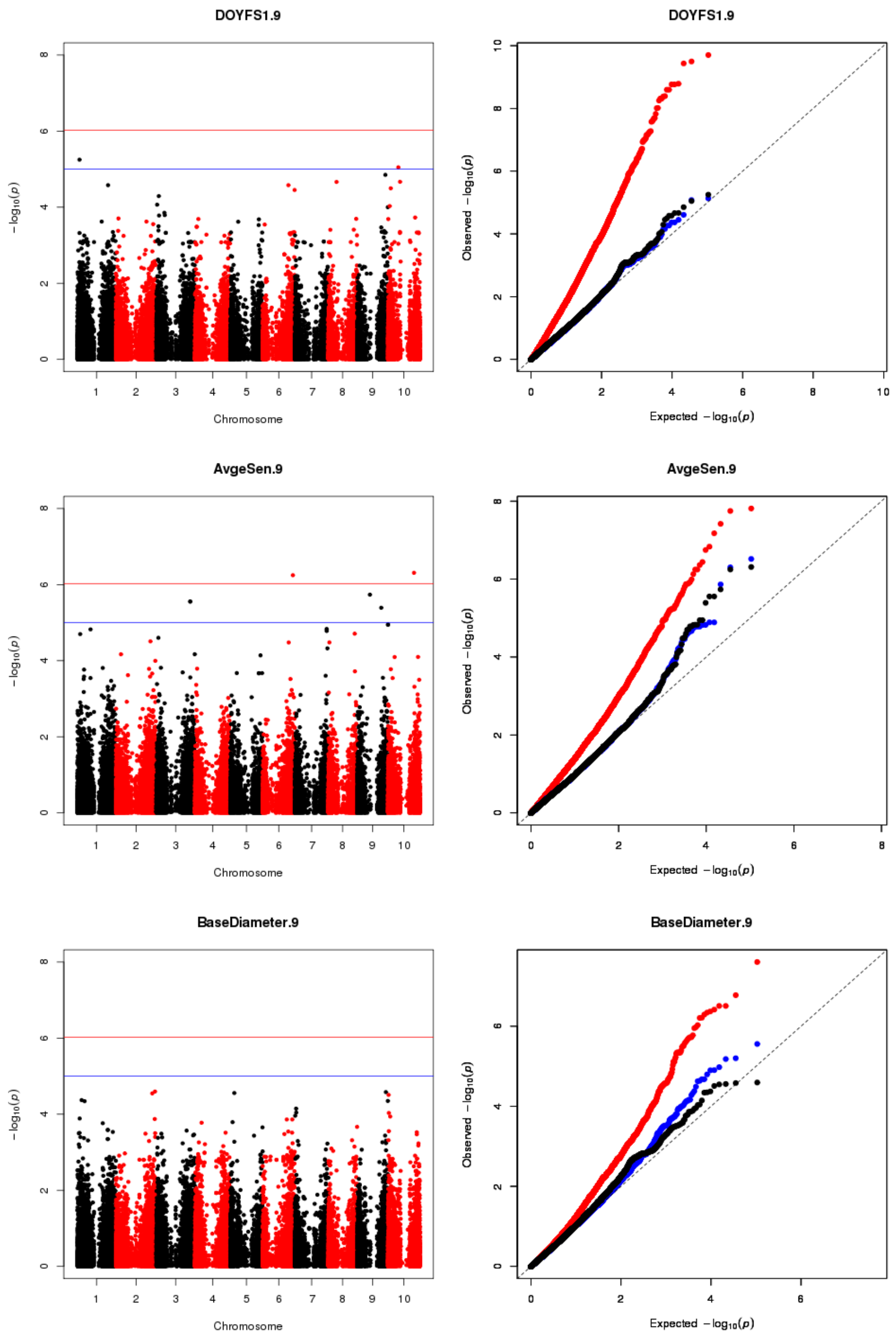

Figure S4 Genome-wide association study (GWAS) results for 17 phenotypic traits in a population of 138 *M. sinensis* genotypes based on 53,174 single-nucleotide variants (SNVs) detected using alignments to the *Sorghum bicolor* genome and filtered using 'liberal' criteria (Table 2). Manhattan (left) and quantile-quantile (QQ) plots (right) are based on the GWAS p-values of all SNVs aligning to *Sorghum* chromosomes 1-10. Manhattan plots: blue lines indicate suggestive ($P = 10^{-5}$) and red lines Bonferroni-adjusted genome-wide significance ($P = 0.05/53{,}174 \approx 9.4 \times 10\text{-}7$) based on mixed linear models including both the identity-by-state (IBS) kinship matrix and the first two eigenvectors (PC1 and PC2) from individual-based principal component analysis. QQ plots: results from analyses including (1) the IBS matrix, as well as PC1 and PC2 (black points); (2) the IBS matrix only (blue points); and (3) simple linear regression without corrections for population structure and relatedness (red points). In the absence of confounding, the majority of points are expected to fall on the x = y diagonal (dashed line).

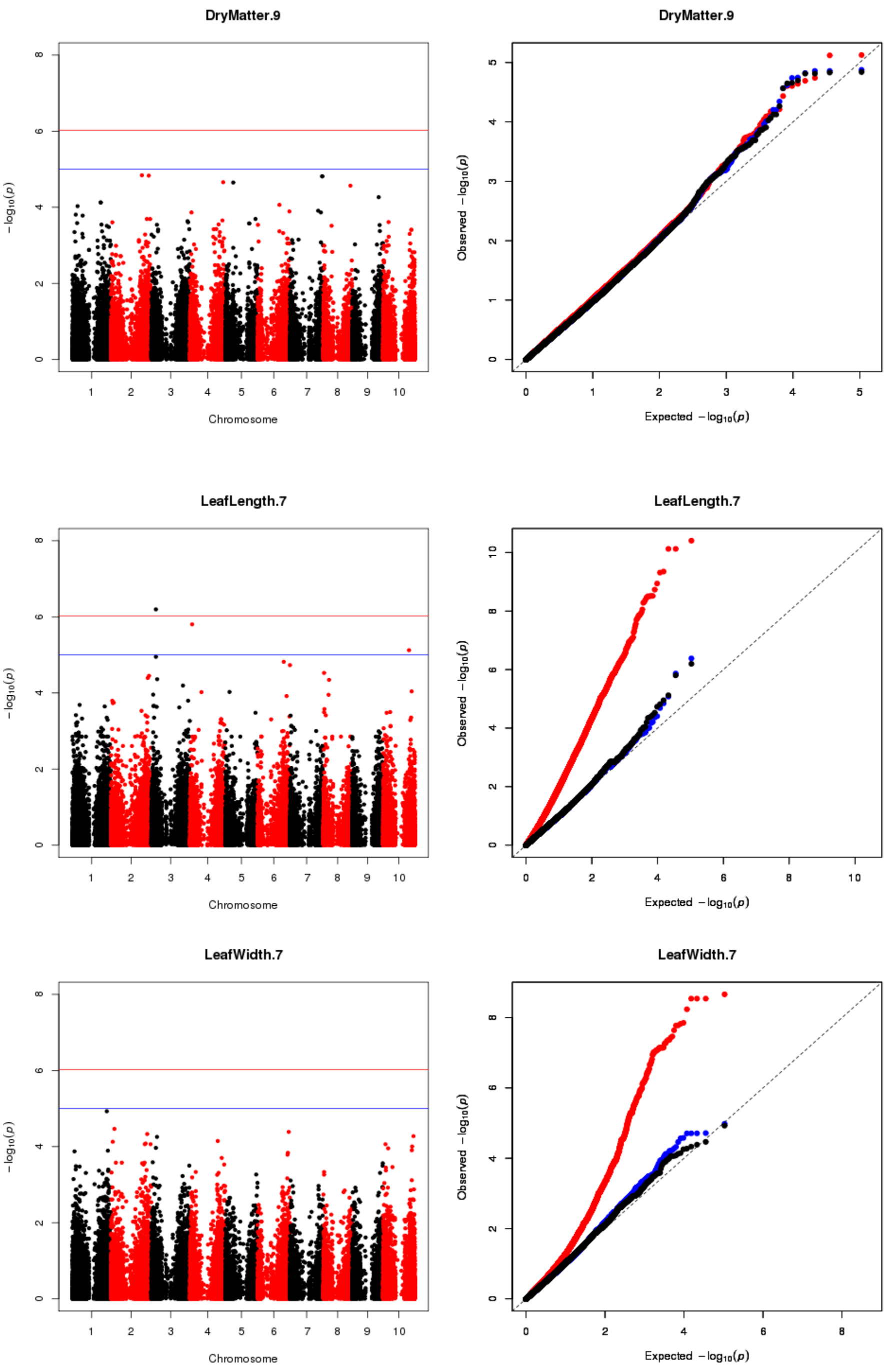

Figure S4 (Continued)

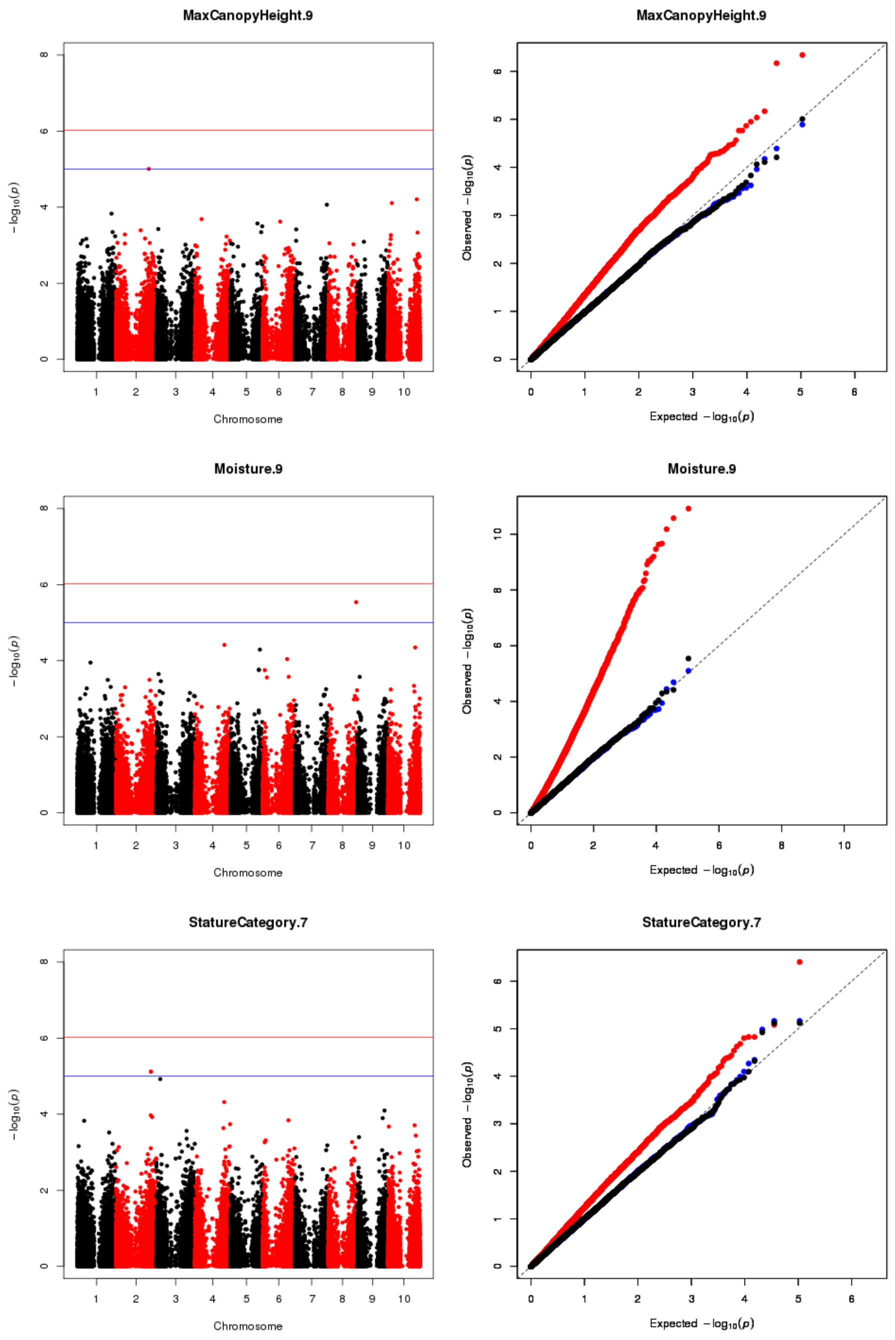

Figure S4 (Continued)

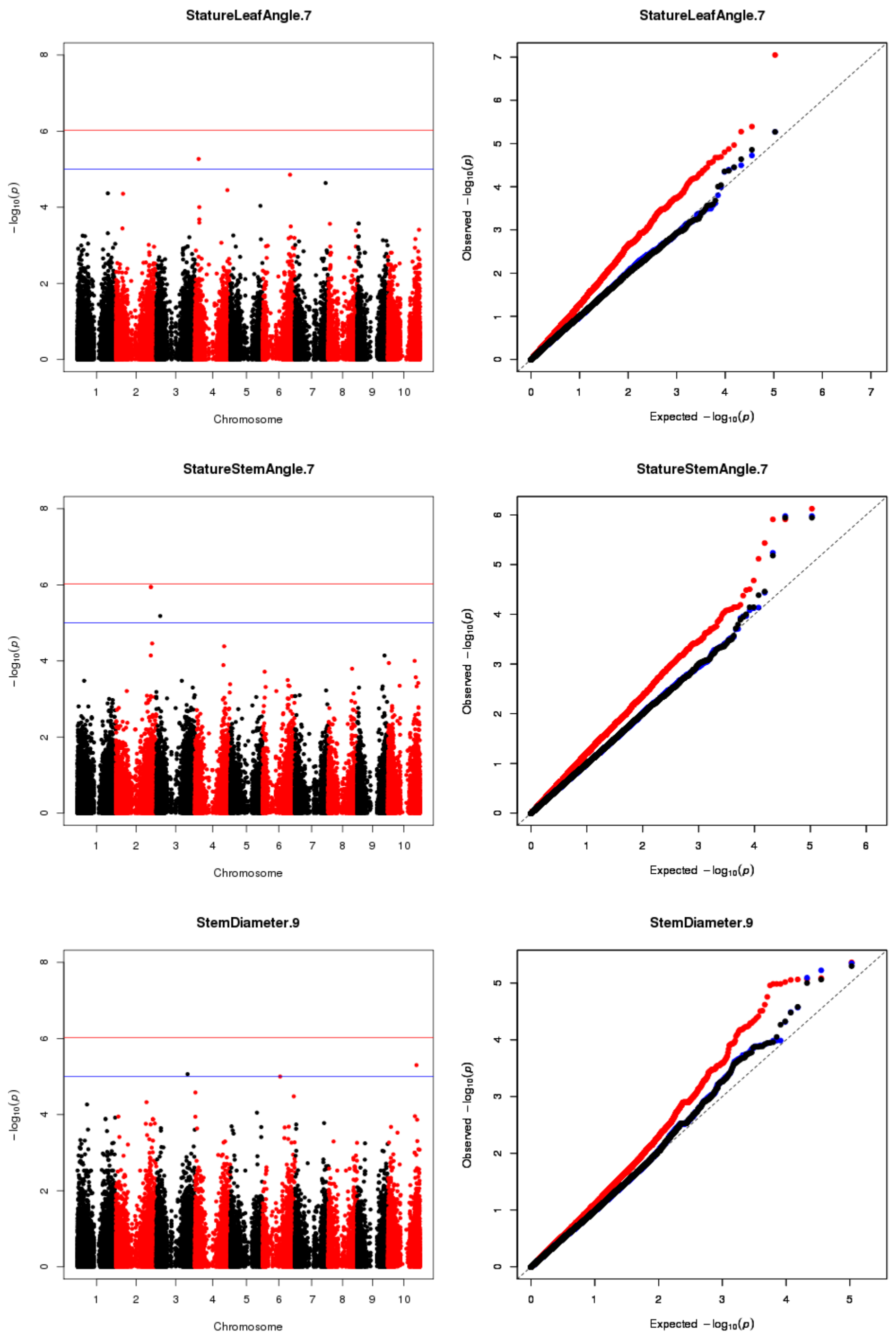

Figure S4 (Continued)

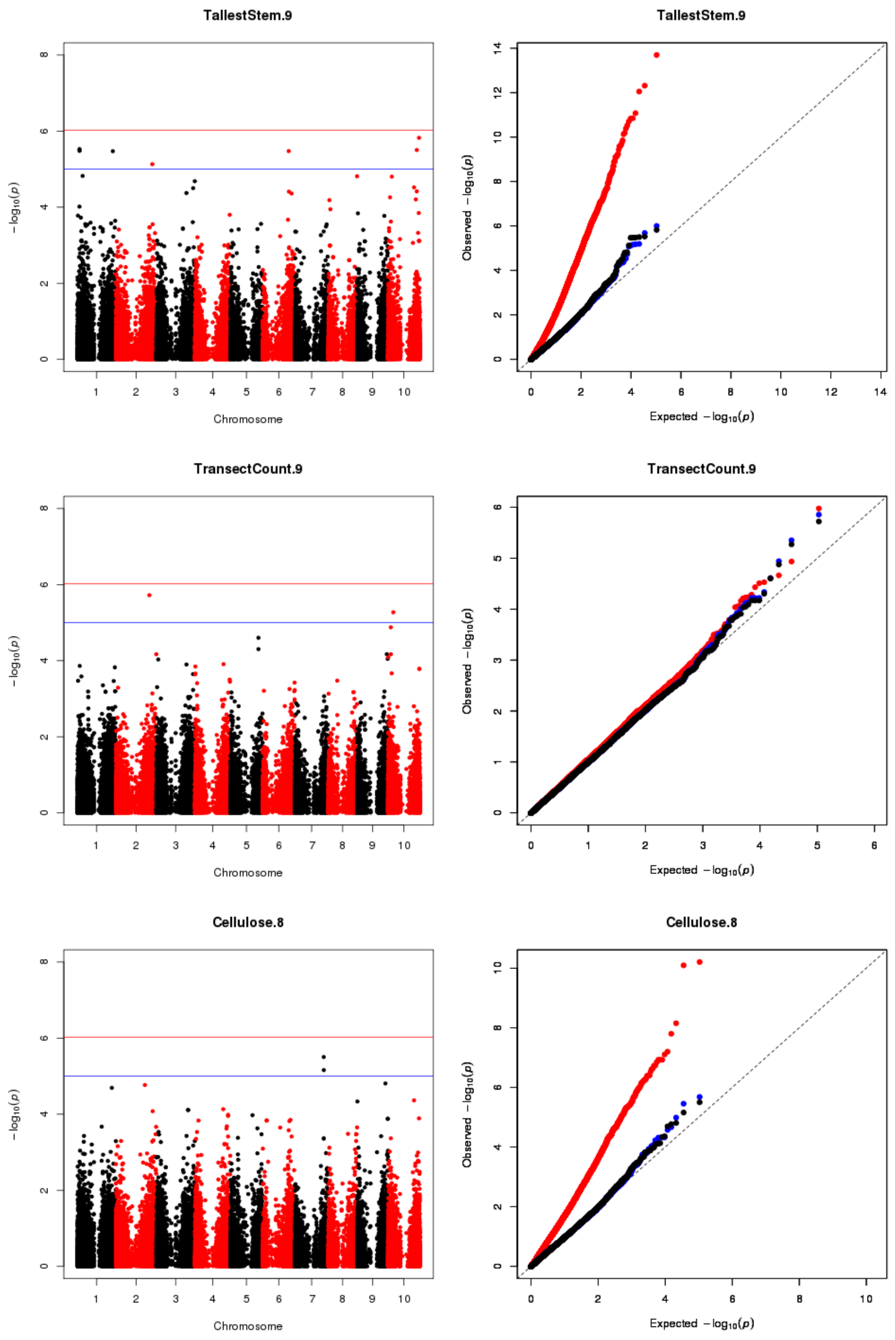

Figure S4 (Continued)

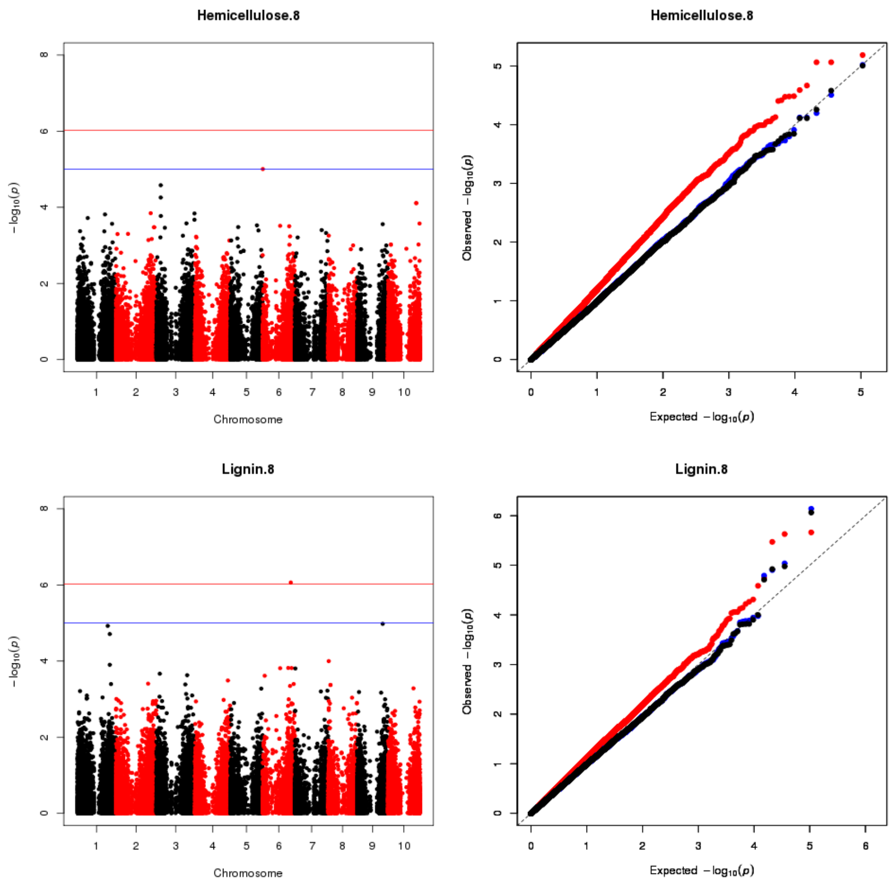

Figure S4 (Continued)

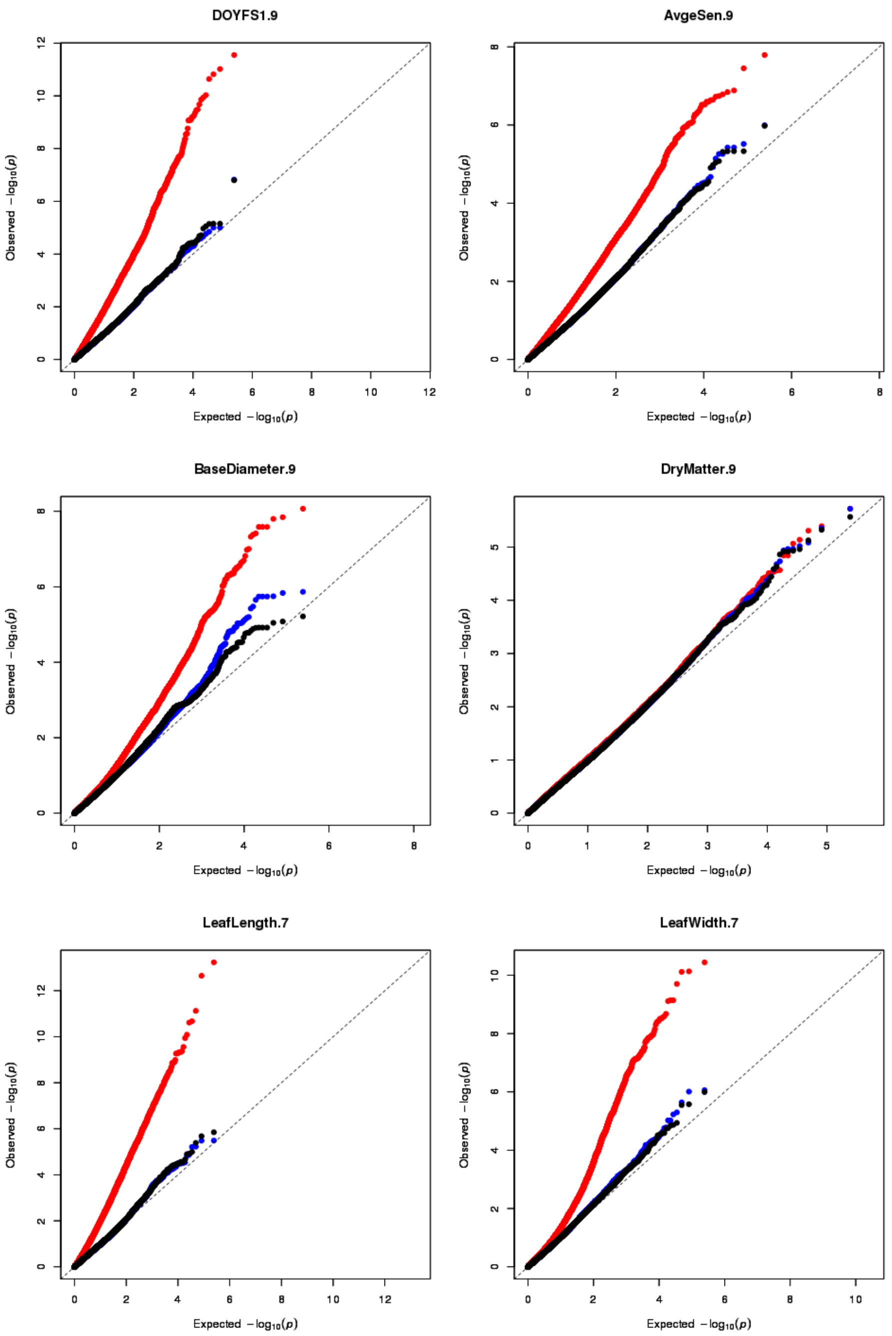

Figure S5 Genome-wide association study (GWAS) results for 17 phenotypic traits in a population of 138 *M. sinensis* genotypes based on 121,771 single-nucleotide variants (SNVs) detected using alignments to a *M. sinensis* pseudo-reference and filtered using 'liberal' criteria (Table 2). Quantile-quantile (QQ) plots are based on the GWAS p-values of all SNVs from analyses including (1) the identity-by-state (IBS) kinship matrix and the first two eigenvectors (PC1 and PC2) from individual-based principal component analysis (black points); (2) the IBS matrix only (blue points); and (3) simple linear regression without corrections for population structure and relatedness (red points). The threshold for Bonferroni-adjusted genome-wide significance was $P = 0.05/121{,}771 \approx 4.1 \times 10^{-7}$. In the absence of confounding, the majority of points are expected to fall on the x = y diagonal (dashed line).

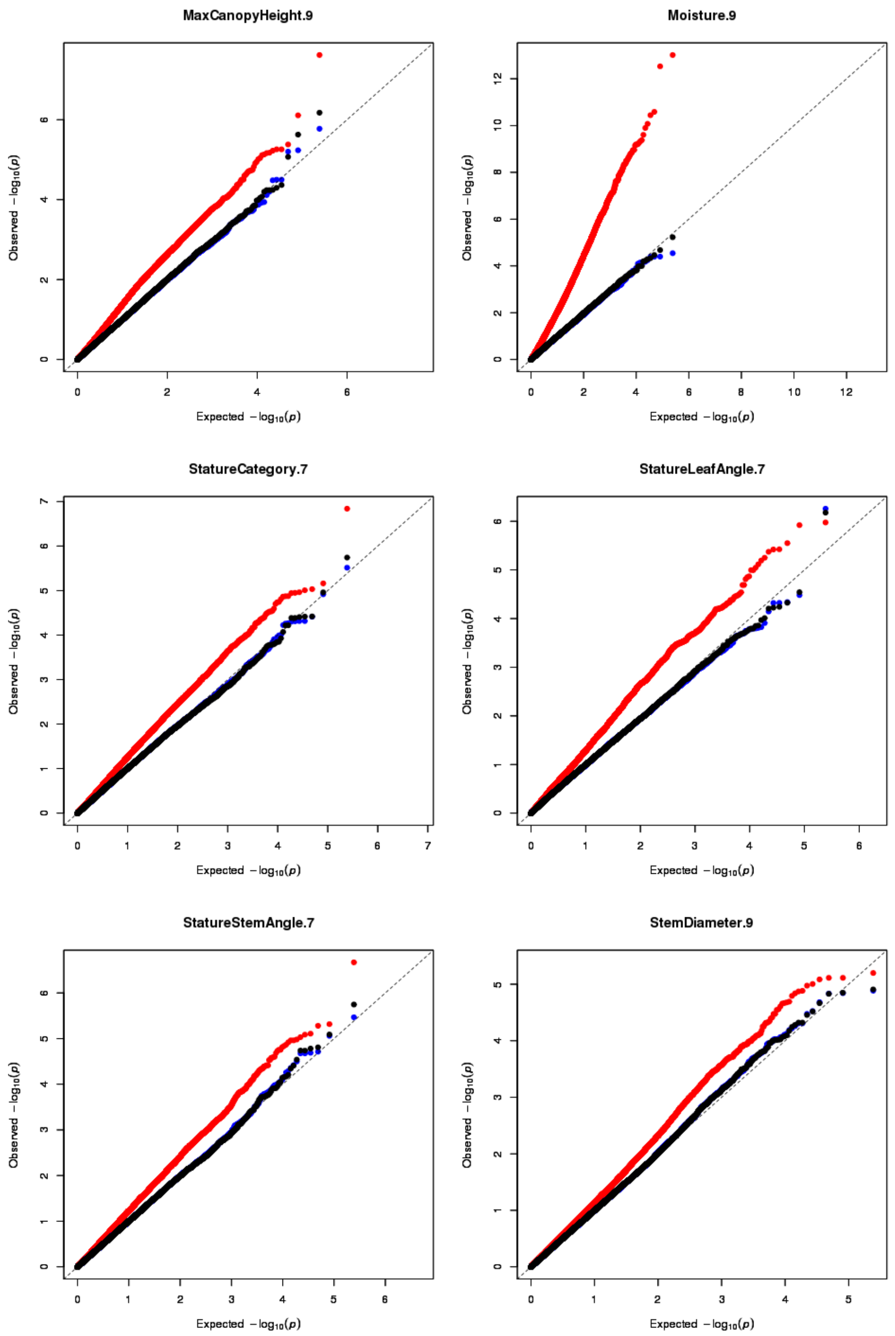

Figure S5 (Continued)

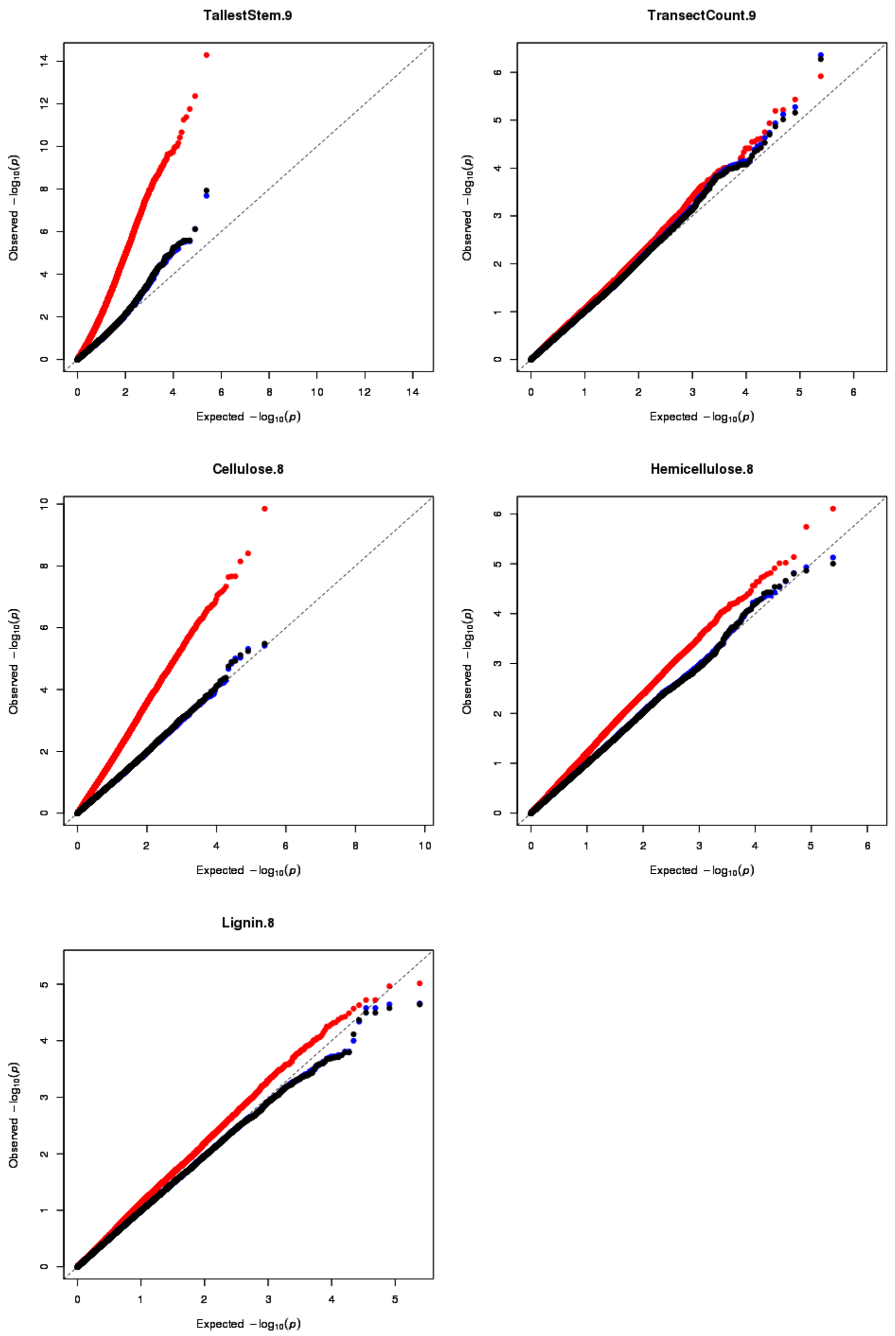

Figure S5 (Continued)

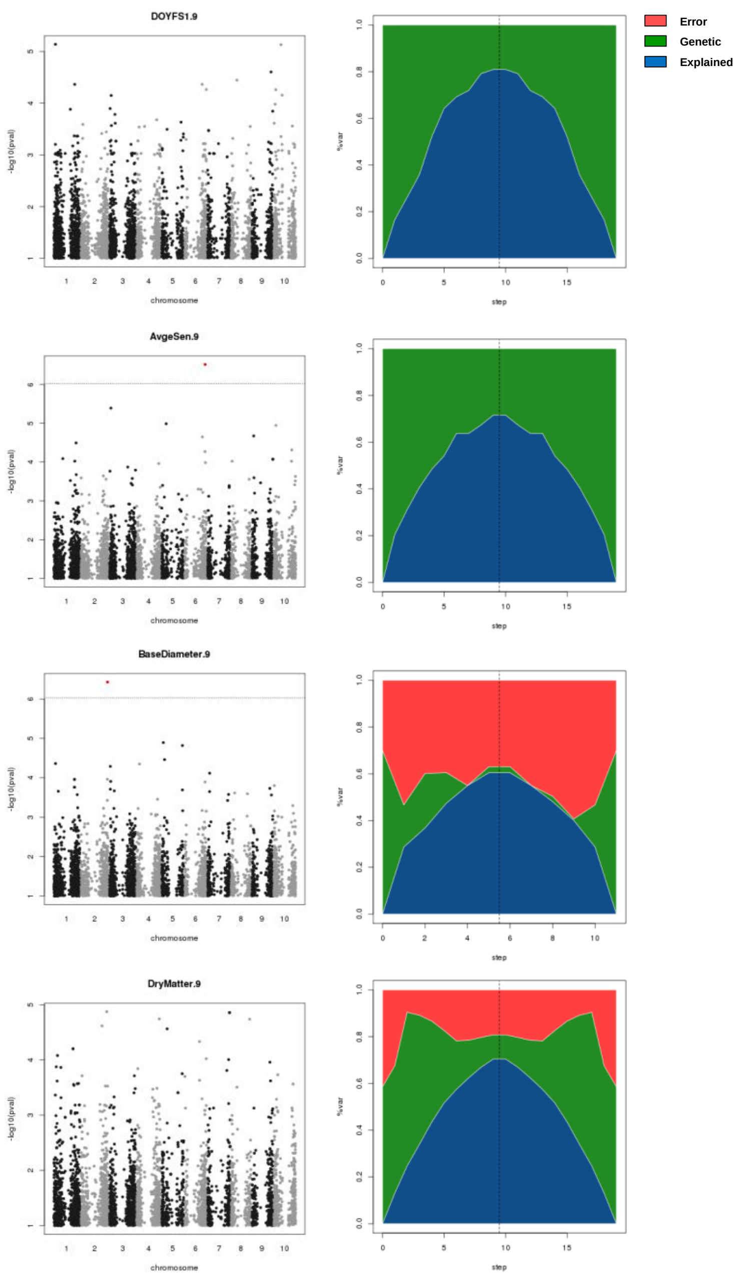

Figure S6 Multi-locus mixed-model (MLMM, Segura *et al.*, 2012) genome-wide association study (GWAS) results for 17 phenotypic traits in a population of 138 *M. sinensis* genotypes based on 53,174 single-nucleotide variants (SNVs) detected using alignments to the *Sorghum bicolor* genome and filtered using 'liberal' criteria (Table 2). Manhattan (left) and variance component plots (right) are based on the MLMM-GWAS p-values of all SNVs aligning to *Sorghum* chromosomes 1-10. Manhattan plots: results from the optimal model selection step according to the multiple Bonferroni criterion; dotted lines indicate Bonferroni-adjusted genome-wide significance ($P = 0.05/53,174 \approx 9.4 \times 10\text{-}7$). Variance component plots: in each step of the model selection procedure, the total variance of trait best linear unbiased predictors (BLUPs) was partitioned into (1) components explained by the individual SNVs included in the model (Explained); (2) components explained by the kinship matrix (Genetic); and (3) residual components (Error). The dotted line indicates the transition between forward selection and backward elimination of SNVs in the model.

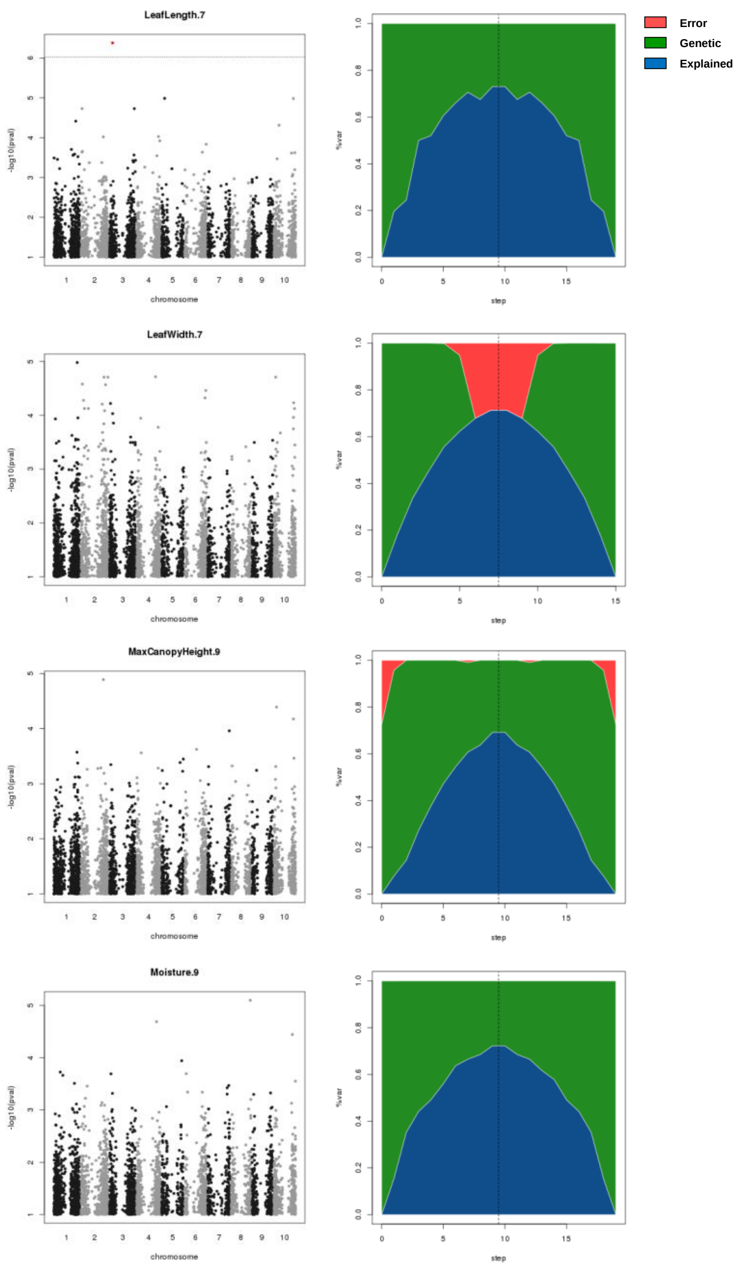

Figure S6 (Continued)

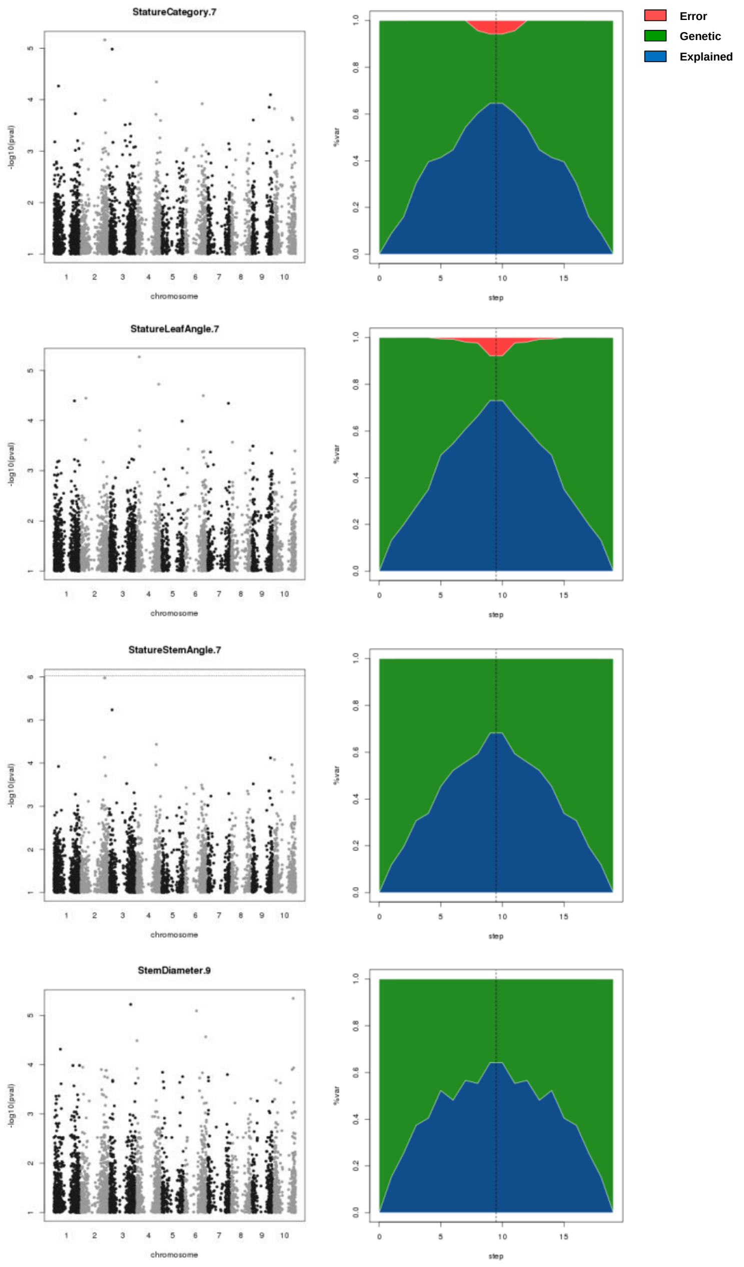

Figure S6 (Continued)

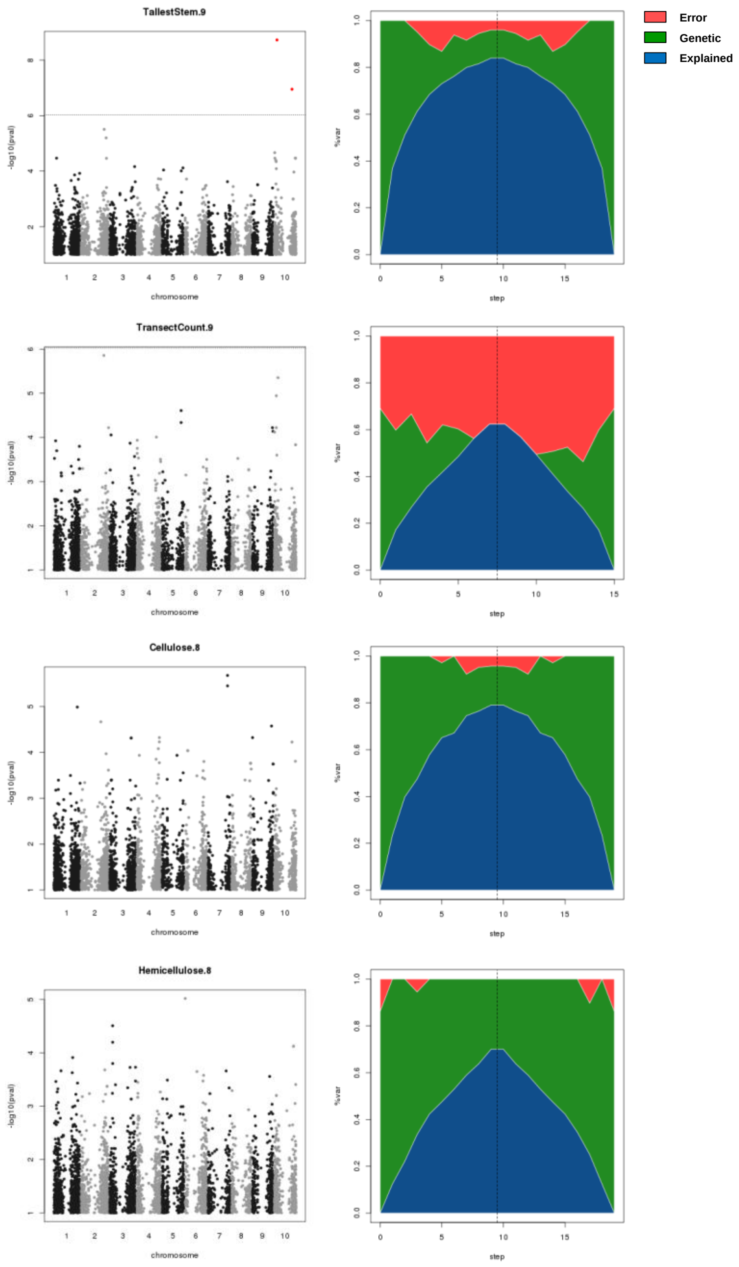

Figure S6 (Continued)

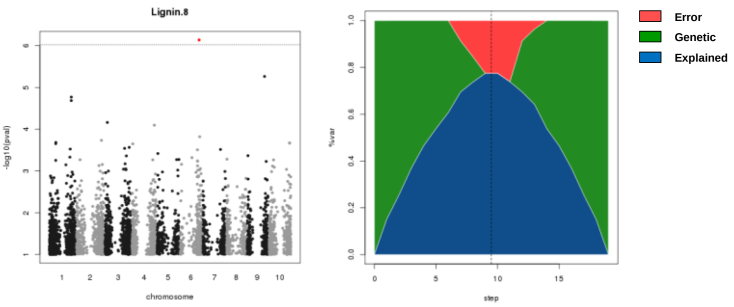

Figure S6 (Continued)

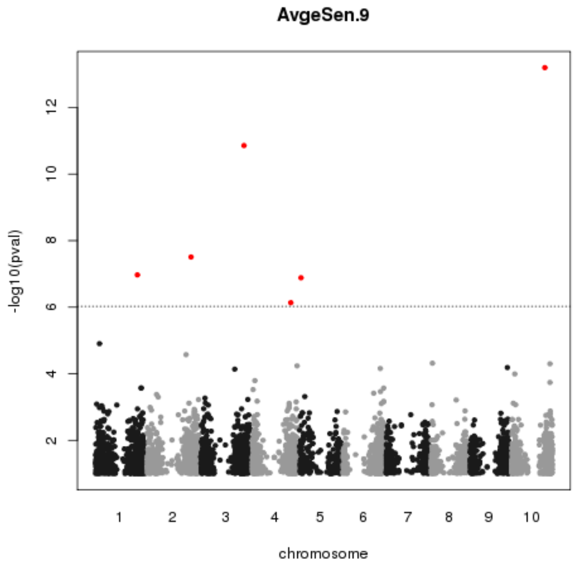

Figure S7 Covariate multi-locus mixed-model (MLMM, Segura *et al.*, 2012) genome-wide association study (GWAS) results for average senescence score (*AvgeSen.9*, Table 1) in a population of 138 *M. sinensis* genotypes based on 53,174 single-nucleotide variants (SNVs) detected using alignments to the *Sorghum bicolor* genome and filtered using 'liberal' criteria (Table 2). In addition to the kinship matrix, MLMMs also included the first two eigenvectors of population structure (Fig. 1) as fixed effects. Only results from the optimal model selection step are shown (multiple Bonferroni criterion); the dotted line indicates Bonferroni-adjusted genome-wide significance (P = 0.05/53,174 ≈ 9.4 × 10-7). Results for other traits were similar or more conservative than those based on MLMMs without population structure covariates (Fig. S6).

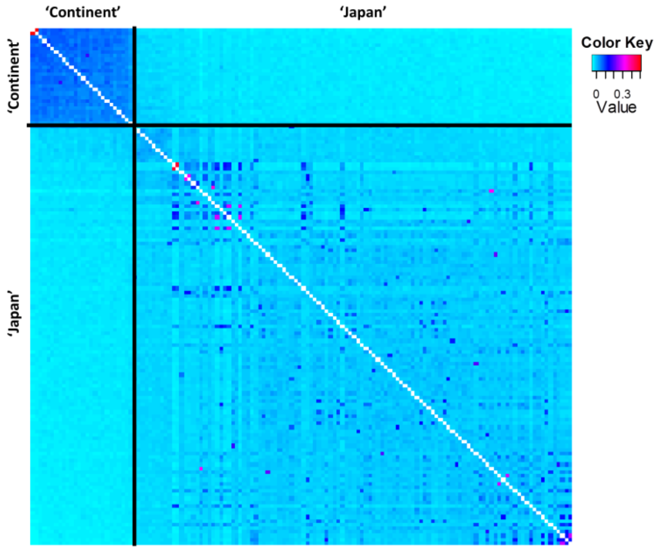

Figure S8 Genetic relationship matrix (Yang *et al.*, 2010, 2011) among 138 *M. sinensis* genotypes based on 20,052 single-nucleotide variants detected using alignments to chromosomes 1-10 of the *Sorghum bicolor* genome and filtered using 'stringent' criteria (Table 2).

Table S1 Simple linear regression measures of performance of genome-wide prediction in a population of 138 *M. sinensis* genotypes based on markers filtered using liberal criteria (Table 2). All regression coefficients are based on 100 random ten-fold cross-validations (i.e., using a training population with *N* = 124 genotypes).

Trait[a]	$H^{2,b}$	$b0_{Sorg}$ (SD)	$b1_{Sorg}$ (SD)	$b0_{Misc}$ (SD)	$b1_{Misc}$ (SD)
**Phenology**					
DOYFS1.9	0.89	1.27 (0.39)	1.35 (0.02)	1.30 (0.37)	1.27 (0.02)
AvgeSen.9	0.83	-0.01 (0.01)	1.49 (0.03)	-0.01 (0.01)	1.35 (0.03)
**Morphology/Biomass**					
BaseDiameter.9	0.52	-0.07 (0.43)	0.95 (0.15)	0.03 (0.38)	0.98 (0.14)
DryMatter.9	0.54	-0.30 (1.35)	0.37 (0.31)	-0.29 (1.45)	0.26 (0.37)
LeafLength.7	0.65	0.13 (0.10)	1.23 (0.02)	0.10 (0.10)	1.17 (0.02)
LeafWidth.7	0.64	0.01 (0.00)	1.29 (0.07)	0.01 (0.00)	1.21 (0.05)
MaxCanopyHeight.9	0.77	0.09 (0.18)	0.96 (0.09)	0.18 (0.15)	0.92 (0.09)
Moisture.9	0.59	0.00 (0.04)	1.27 (0.02)	0.00 (0.03)	1.23 (0.01)
StatureCategory.7	0.48	0.00 (0.01)	1.11 (0.06)	0.00 (0.01)	1.06 (0.05)
StatureLeafAngle.7	0.50	0.00 (0.00)	1.17 (0.07)	0.00 (0.00)	1.08 (0.06)
StatureStemAngle.7	0.48	0.00 (0.00)	1.08 (0.06)	0.00 (0.00)	1.04 (0.06)
StemDiameter.9	0.60	0.02 (0.01)	1.72 (0.10)	0.02 (0.01)	1.40 (0.06)
TallestStem.9	0.88	0.06 (0.38)	1.10 (0.02)	0.29 (0.33)	1.04 (0.02)
TransectCount.9	0.51	-0.03 (0.03)	0.82 (0.23)	-0.02 (0.05)	1.16 (0.16)
**Cell wall composition**					
Cellulose.8	0.79	-0.02 (0.02)	1.23 (0.03)	-0.02 (0.02)	1.15 (0.03)
Hemicellulose.8	0.60	0.00 (0.01)	0.84 (0.11)	0.00 (0.00)	0.72 (0.16)
Lignin.8	0.66	0.00 (0.01)	1.36 (0.07)	0.00 (0.00)	0.95 (0.07)
**Average**	**0.64**	**0.07 (0.32)**	**1.14 (0.30)**	**0.09 (0.33)**	**1.06 (0.26)**

[a] Trait: phenotypic trait as defined in Table 1.
[b] H^2: broad-sense heritability (see Materials and Methods).
[c] $b0_{Sorg}$ *(SD)*: average intercept of simple linear regression of best linear unbiased predictors (BLUPs) calculated from field data on those estimated using ridge regression based on 53,174 single-nucleotide variants (SNVs) obtained through alignments to the *Sorghum bicolor* genome. Standard deviations across the 100 random ten-fold cross-validations are shown in parentheses.
[d] $b1_{Sorg}$ *(SD)*: average slope of simple linear regression of BLUPs calculated from field data on those estimated using ridge regression based on 53,174 SNVs obtained through alignments to the *Sorghum bicolor* genome. Standard deviations across the 100 random ten-fold cross-validations are shown in parentheses.
[e] $b0_{Misc}$ *(SD)*: average intercept of simple linear regression of BLUPs calculated from field data on those estimated using ridge regression based on 121,771 SNVs obtained through alignments to a *M. sinensis* pseudo-reference. Standard deviations across the 100 random ten-fold cross-validations are shown in parentheses.
[f] $b1_{Misc}$ *(SD)*: average slope of simple linear regression of BLUPs calculated from field data on those estimated using ridge regression based on 121,771 SNVs obtained through alignments to a *M. sinensis* pseudo-reference. Standard deviations across the 100 random ten-fold cross-validations are shown in parentheses.
[g] Average (SD): overall average and standard deviation across traits.

Method S1 *Miscanthus* pseudo-reference

First, we selected a reference genotype based on its relatively large sequence coverage (17,152,027 reads). After trimming reads from the 3' end to a total length of 85 bp, custom Perl scripts were used to cluster identical sequences represented between 30 and 250 times. Based on the observed coverage distribution, these sequences were assumed to represent low- or single-copy restriction-site associated DNA (RAD) loci in the *Miscanthus* genome. As a result of these procedures, 149,974 RAD clusters were coalesced from 8,323,912 sequence reads (i.e., approximately 49% of the total sequence data). The assembly was then condensed to *fasta* format and aligned to itself using BWA (Li & Durbin, 2009), with the following command options: *bwa aln –n 5 –N*. This self-alignment was used to identify highly similar sequences within the assembly. Any cluster with more than two observed presumed haplotypes was discarded. This was done to eliminate duplicated sequences in the assembly. The final filtered assembly contained 48,426 RAD clusters, representing 4.1 Mb of putatively low-copy *Miscanthus* genomic sequence.

References

Evanno G, Regnaut S, Goudet J. 2005. Detecting the number of clusters of individuals using the software STRUCTURE: a simulation study. *Molecular Ecology* **14**(8): 2611-2620.

Falush D, Stephens M, Pritchard JK. 2003. Inference of population structure using multilocus genotype data: Linked loci and correlated allele frequencies. *Genetics* **164**(4): 1567-1587.

Falush D, Stephens M, Pritchard JK. 2007. Inference of population structure using multilocus genotype data: dominant markers and null alleles. *Molecular Ecology Notes* **7**(4): 574-578.

Jakobsson M, Rosenberg NA. 2007. CLUMPP: a cluster matching and permutation program for dealing with label switching and multimodality in analysis of population structure. *Bioinformatics* **23**(14): 1801-1806.

Li H, Durbin R. 2009. Fast and accurate short read alignment with Burrows-Wheeler transform. *Bioinformatics* **25**(14): 1754-1760.

Patterson N, Price AL, Reich D. 2006. Population structure and eigenanalysis. *PLOS Genetics* **2**(12): 2074-2093.

Pritchard JK, Stephens M, Donnelly P. 2000. Inference of population structure using multilocus genotype data. *Genetics* **155**(2): 945-959.

Rosenberg NA. 2004. DISTRUCT: a program for the graphical display of population structure. *Molecular Ecology Notes* **4**(1): 137-138.

Segura V, Vilhjalmsson BJ, Platt A, Korte A, Seren U, Long Q, Nordborg M. 2012. An efficient multi-locus mixed-model approach for genome-wide association studies in structured populations. *Nature Genetics* **44**(7): 825-U144.

Yang J, Lee SH, Goddard ME, Visscher PM. 2011. GCTA: A tool for genome-wide complex trait analysis. *American Journal of Human Genetics* **88**(1): 76-82.

Yang JA, Benyamin B, McEvoy BP, Gordon S, Henders AK, Nyholt DR, Madden PA, Heath AC, Martin NG, Montgomery GW, Goddard ME, Visscher PM. 2010. Common SNPs explain a large proportion of the heritability for human height. *Nature Genetics* **42**(7): 565-U131.

Yu JM, Pressoir G, Briggs WH, Bi IV, Yamasaki M, Doebley JF, McMullen MD, Gaut BS, Nielsen DM, Holland JB, Kresovich S, Buckler ES. 2006. A unified mixed-model method for association mapping that accounts for multiple levels of relatedness. *Nature Genetics* **38**(2): 203-208.
